# Colon Cancer Diagnosis Based on Machine Learning and Deep Learning: Modalities and Analysis Techniques

**DOI:** 10.3390/s22239250

**Published:** 2022-11-28

**Authors:** Mai Tharwat, Nehal A. Sakr, Shaker El-Sappagh, Hassan Soliman, Kyung-Sup Kwak, Mohammed Elmogy

**Affiliations:** 1 Information Technology Department, Faculty of Computers and Information, Mansoura University, Mansoura 35516, Egypt; 2 Information Systems Department, Faculty of Computers and Artificial Intelligence, Benha University, Benha 13512, Egypt; 3 Faculty of Computer Science and Engineering, Galala University, Suez 435611, Egypt; 4 Department of Information and Communication Engineering, Inha University, Incheon 22212, Republic of Korea

**Keywords:** colon cancer diagnosis, imaging modalities, deep-learning techniques, histopathology image analysis, medical image analysis

## Abstract

The treatment and diagnosis of colon cancer are considered to be social and economic challenges due to the high mortality rates. Every year, around the world, almost half a million people contract cancer, including colon cancer. Determining the grade of colon cancer mainly depends on analyzing the gland’s structure by tissue region, which has led to the existence of various tests for screening that can be utilized to investigate polyp images and colorectal cancer. This article presents a comprehensive survey on the diagnosis of colon cancer. This covers many aspects related to colon cancer, such as its symptoms and grades as well as the available imaging modalities (particularly, histopathology images used for analysis) in addition to common diagnosis systems. Furthermore, the most widely used datasets and performance evaluation metrics are discussed. We provide a comprehensive review of the current studies on colon cancer, classified into deep-learning (DL) and machine-learning (ML) techniques, and we identify their main strengths and limitations. These techniques provide extensive support for identifying the early stages of cancer that lead to early treatment of the disease and produce a lower mortality rate compared with the rate produced after symptoms develop. In addition, these methods can help to prevent colorectal cancer from progressing through the removal of pre-malignant polyps, which can be achieved using screening tests to make the disease easier to diagnose. Finally, the existing challenges and future research directions that open the way for future work in this field are presented.

## 1. Introduction

Colon cancer is a specific kind of tumor that originates in the colon or the rectum, existing in the digestive system at the lower portion of [1]. The colon forms the main part of the large intestine, and the rectum exists at the end of the colon [2]. Colon cancer is considered to be one of the leading causes of death in the industrialized and Western world, and its incidence grown [3]. In 2012, about 1.4 million people were diagnosed with this disease. In 2017, there were almost 50,260 deaths reported [4]. The main reasons for incidence stem from unhealthy habits, including chain-smoking and eating high amounts of red meat and little fruit in addition to a family history of disease and increasing age [5].

**Figure 1:**
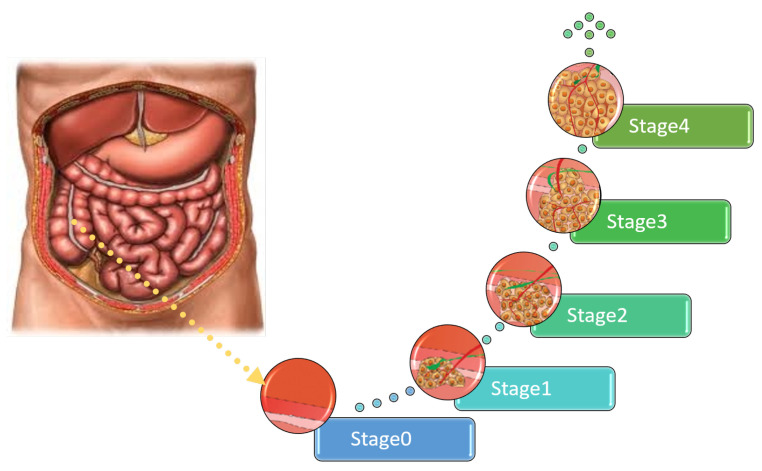
The different stages of colon cancer.

There are four main grades of colon cancer as shown in Figure 1 [6]. The first stage is defined as the mucosa or lining of the colon or rectum, while the organ wall has not yet developed tumors. In the second stage, the walls of the rectum or colon begin to develop tumors; however, nearby tissues or lymph nodes are not yet affected [7].

The third stage is reached when the tumor has spread only to the lymph tissues but has not yet spread to any other body part. In the fourth stage, the tumor spreads to other organs, such as the lungs [8]. The prevalence in stage four has different symptoms, depending on the organ to which the tumor has spread as shown in Table 1 [9].

Although colorectal cancer does not have apparent symptoms, particularly in its early stages [10], there are unusual symptoms, such as abdominal pain, constipation, excess gas, diarrhea, and changes in the color and shape of stool (e.g., narrow stool, abdominal cramps, and blood in the stool) [11]. According to ACS, the most common reason for colon cancer stems from adenocarcinoma disorders, accounting for almost 96% of all stages of this type of cancer [12].

Colorectal cancers can also arise from other tissues that have tumors, such as carcinomas that first arise in the hormone-producing polyps of the intestines [13] and lymphomas that may first form in the colon; however, this is less common. These sarcomas start in small tissues, such as gastrointestinal stromal tumors that start as normal tumors and later become cancerous (these at a few times begin in the colon but almost start in the digestive tract) [14].

Not all types of tumors are malignant. There is a non-spreadable or benign type that is not fatal or destructive as the spreadable type is. The difference of biological tumor structures presents great challenges for automatic and manual analysis of histopathological images (HIs) [15]. A manual examination of the cancer level/grade relies on the pathologist’s visual assessment, which is subjective, time-consuming, and potentially error-prone [16]. An incorrect or late diagnosis can cause anxiety for many patients. Therefore, Medical Image Analysis (MIA) is required to process and analyze HIs automatically. Such an MIA system can be used to classify colon cancer and present an objective, and accurate assessment of various grades of this cancer [17].

A diagnosis of colon cancer can be implemented automatically with the power of AI, leading to more types of diagnosis with less cost and in less time. AI-based diagnosis methods can be categorized into ML techniques and DL techniques. Recent advances in digital image processing (DIP) techniques and DL play an essential role in the diagnostic process [18]. In this paper, we show a comprehensive survey on different ML and DL techniques proposed for identifying the different stages of colon cancer. This can be accomplished using different imaging modalities. However, we focus on histopathological imaging, which is considered the best modality used to examine, classify, locate and provide a comprehensive view of the different cancer stages.

Due to the high mortality rates caused by colon cancer and the power of AI-based techniques for early diagnosis, many studies have been proposed on colon cancer diagnosis. However, the number of surveys presented in this research topic is limited. Starting from 2009, C. Demir and B. Yener [19] reported a brief review on detection of colon cancer on the basis of histopathological images. Then, in 2013, Rathore et al. [6] introduced a review on techniques of detection of colon cancer that were classified according to the employed dataset and methodology into biopsy-based analysis methods and physical sample analysis methods.

In this study, the authors claimed that much more work is required in their experiments regarding the performance measures and parameter tuning. In 2020, Pacal et al. [20] presented a comprehensive survey on the application of DL to colon cancer diagnosis. This work gives a detailed discussion on DL identifying its basic architectures and emerging topics and then summarizes the recent DL-related studies. Finally, in 2022, Davri et al. [21] published a systematic review on colon cancer diagnosis using histopathological images. They investigated the application of DL techniques for cancer diagnosis from both the medical and technical viewpoints.

In this review, the authors presented a summary of recent DL-based methods. Therefore, a detailed analysis and comparison of these techniques illustrating their working methodology, strengths, and limitations is still required. As noticed, recent reviews focused only on the application of DL for colon cancer diagnosis. Moreover, a detailed analysis of these studies is required, identifying their limitations to fulfill the main need of reviewers who are in this field by improving the open research directions.

The main purpose of this survey is to discuss and summarize the recent research attempts proposed for colon cancer identification and diagnosis, classified into ML-based and DL-based models. First, we discus many aspects related to colon cancer diagnosis, such as the existing imaging modalities used for analysis with special attention on histopathology images, in addition to the common cancer diagnosis systems. We review and compare the available datasets for colon cancer apart from the common performance evaluation metrics. Then, we review the existing work, identifying its working methodology in addition to the strengths and limitations. Finally, we discuss the most obvious challenges related to the automatic diagnosis of colon cancer to open the mind for future research directions.

The rest of this survey is categorized into eight parts as follows. Section 2 shows the methodology followed to present this survey, such as the keywords used for the search, the data sources, the criteria used for the inclusion and exclusion of articles, and the selection of articles. Section 3 discusses different aspects related to colon cancer diagnosis, such as the different screening tests used to analyze this type of cancer, the common diagnosis systems that are based on the analysis of HIs, the available datasets, and common performance evaluation metrics.

**Figure 2:**
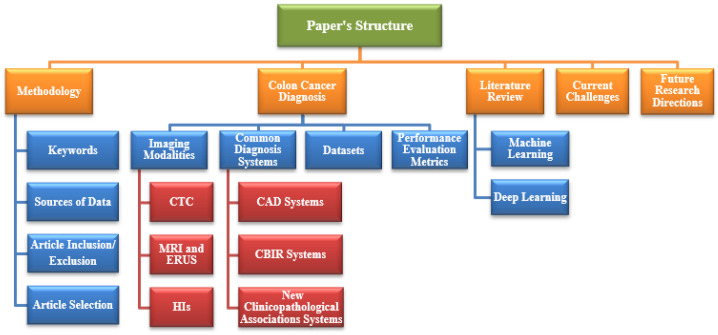
The structure of the survey.

Section 4 presents a literature review of the conventional ML and DL techniques proposed for colon cancer diagnosis using different modalities. Section 5 presents the existing challenges discovered thus far, and Section 6 presents the future research directions to obtain better techniques. Finally, Section 7 concludes this study. Figure 2 shows the structure of this survey. Table 2 presents a list of abbreviations with the corresponding definitions used in this survey.

## 2. Research Methodology

**Figure 3:**
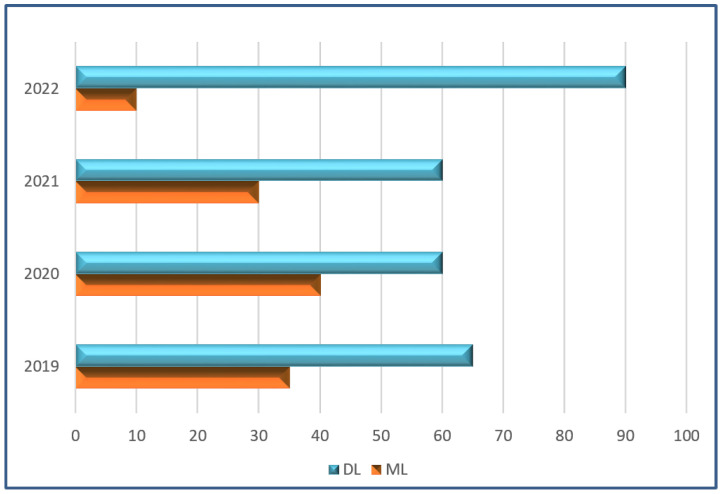
Frequency-based analysis of technique types in percentages.

**Figure 4:**
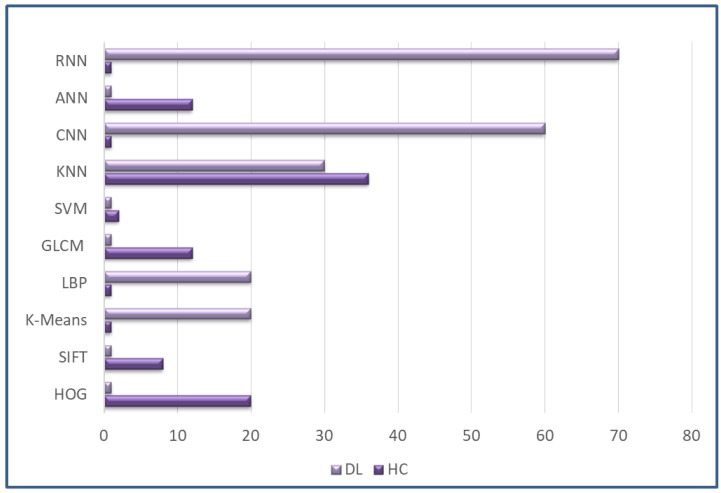
Frequency-based analysis of sub-technique types in percentages.

In this part, we present the approach used to survey the modalities and techniques applied to diagnose colon cancer from the year 2019 to 2022. We discuss the keywords used for the search, the sources of data, the exclusion/inclusion criteria of articles, and the principles of article selection. Figure 3 shows the analysis frequency of DL and ML techniques. Figure 4 represents sub-techniques used in detection of colon cancer, classified into deep-learning (DL) and hand-crafted (HC) Techniques.

### 2.1. Keywords

Initially, we used specific keywords close to colon cancer in the search process, such as ’colon cancer diagnosis’ and ’colorectal cancer’. After the search, new words were collected from the resulting articles to obtain numerous keywords. Furthermore, new keywords, such as ’Imaging Modalities’, ’Deep Learning Techniques’, ’Histopathology Image Analysis’, and ’Medical Image Analysis’ were used based on our understanding of the research topic.

### 2.2. Data Sources

Various academic databases were used for obtaining relevant articles for the survey as shown in Table 3.

### 2.3. Inclusion and Exclusion of Article Criteria

**Figure 5:**
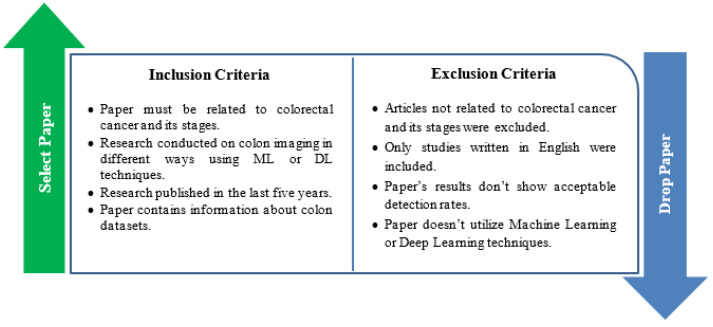
Inclusion and exclusion criteria.

In order to identify the most relevant publications for further review in our study, exclusion/inclusion measures were utilized that depended on our study objective. The studies that matched with the inclusion criteria were considered related to the study, while those that did not match the criteria were eliminated. Figure 5 shows the inclusion and exclusion criteria used in our research.

### 2.4. Selection of The Articles

To select the most significant articles, three stages were followed. First, the article title, abstract, and keywords were checked for relevant articles. The second stage refined the results obtained in the previous stage by analyzing the abstract, introduction, and conclusion of the obtained articles. Finally, the last stage was to read and analyze the main body of the articles more deeply and to determine their relevance to our research.

## 3. Colon Cancer Diagnosis

Before going in depth and reviewing the current work on colon cancer diagnosis, many aspects related to the diagnosis process should be taken into consideration, such as the image modality used, type of diagnosis system, the dataset used, and the metrics used for evaluation. Therefore, in the following subsections, we discuss these aspects.

### 3.1. Imaging Modalities

As mentioned before, our main goal is the automatic diagnosis of colon cancer with high detection accuracy and without manual intervention. In this section, we take an in-depth look at the different imaging modalities recently applied for MIA, including Computed Tomography (CT), Endorectal Ultrasound (ERUS), virtual Computed Tomography Colonoscopy (CTC), and Magnetic Resonance Imaging (MRI) [22] in addition to other modalities, such as Histopathological Imaging (HI) and Positron Emission Tomography (PET) [23]. A brief comparison between the different imaging modalities from different aspects is presented in Table 4.

#### 3.1.1. Virtual Computed Tomography Colonoscopy (CTC)

CTC imaging is the first modality to rely on low-density attenuation X-rays. For accurate examination, the colon/rectum must be adequately inflated using a thin carbon dioxide rectal catheter or by air pushed into the patient’s colon [24]. Air distention of the colon is preferred because of its ease of administration and lower cost. Acquisition of CT can be performed twice for two reasons. The first reason is to better reach the different parts of the colon through gravitational compression, which is based on its abdominal structures.

**Figure 6:**
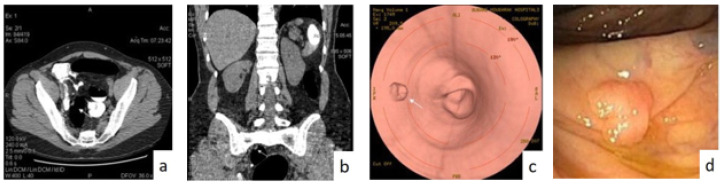
Different images of CT colonoscopy: (**a**) Axial. (**b**) Sagittal images. (**c**) Image of virtual colonoscopy. (**d**) Image colonoscopy showing the polyp with true-positive findings.

The second reason is that the polyps can exist in the intestine walls from fecal or liquid residues in the prone and passive position (or vice versa). Appropriate software can be used to remove residual fluid from CTC images. Different images of the CTC colonoscopy are shown in Figure 6. Horton et al. [25] showed that the CT modality is valuable for the planning of colon cancer surgery since it can capture the regional extension of the tumor in addition to distant metastases and adenopathies. With CT imaging, colorectal cancer usually appears as a soft-tissue mass, a discrete mass, or in a thickened cushion-walled form with intestinal discomfort.

Ding et al. [26] presented a comparison between CTC and colonoscopy for the ability to detect a larger colorectal polyp. Two meta-analysis studies showed the high accuracy of colon cancer CTC detection—a high sensitivity equal to 100%. CTC is an approach to screen patients without actual symptoms as suggested by the ACS as a method for validation of diagnosis since 2008 and is considered the primary method used in screening for CRC [27].

However, Kekelidze et al. [28] used a multi-center randomized trial that included a large sample of patients, almost 1610 patients, and the results showed that this suggestion was revalidated. CTC is a non-surgical replacement to MRI colonoscopy that is not related to radiation exposure but has similar sensitivity [29]. However, CTC is not recommended as a screening modality because the results of the available studies are insufficient.

#### 3.1.2. Magnetic Resonance Imaging (MRI) and Endorectal Ultrasound (ERUS)

The MRI modality is recommended for the early stages of tumors as it can define the location and accurately identify the relationship between the tissues, the reflective Britton, and the total extension [10]. The MRI modality is characterized by its high precision in determining the length of the tumor by measuring the distance between the tumor distal part and the junction of anorectal. On the other hand, the ERUS modality can be used to assess the integrity of the rectal wall layers. Endorectal MRI has many disadvantages, including limited availability, being less patient-friendly, and being costly. Therefore, this method is not recommended by the European society for medical oncology guidelines [30].

Burdan et al. [10] presented an overview of the ERUS and MRI modalities. These modalities have the ability to capture and explore the morphology of the colon in detail. Therefore, they are considered an essential tool in locating the tumor at any stage. Each stage of cancer has its treatment plan. With the ERUS and MRI imaging modalities, it is easy to determine the proper treatment plan for an early-stage tumor faster than other modalities.

**Table 4:** Comparison between different imaging modalities.

No.	Image Modality	Characteristics	Diagnosis Applications	CONS.	PROS.
1	Computed Tomography Colonoscopy (CTC) [31].	To obtain the images uses magnets and radio waves to create [32].	Fractures. Bones. Tumors. Cancer diagnosis. Response and development to treatment.	Less tolerated [10].	More common. Less expensive. Accurate and needs less time to study it. Prefect results in diagnosing, particularly in deceases of the heart lining and the large vessels [10].
2	Magnetic Resonance Imaging (MRI) [33].	Uses radio waves and magnetic [34].	Heart. Ankles. Brain. Wrists. Breasts. Joints. Vessels. Blood.	Needs more time to study it [35]. The size is several hundred megabytes. Confined environment and many noises. It cannot be used with medical devices or foreign devices [10].	Produces more accurate, detailed images. Without using harmful radiation, images can be captured from different angles. Perfect results in diagnosing, particularly in deceases of the heart lining and the large vessels. Used to differentiate external tissue [10].
3	Histopathology Images (HI) [36,37].	Using glass slides and braces on surgical specimens or microscopy to examine a biopsy [38].	Breast. Meningioma. Colon. Prostate.	Needs a different analysis with different tasks of the organs that are visualized using a microscope.	Useful in medical decisions and for studies of biology. Generally used to produce "ground truths" of other modalities of medical images. The size of a histology slide is a few megabytes.
4	Positron Emission Tomography (PET) [39].	Showing activity using a radioactive drug (tracer) [40].	Brain. Cervical. Colo-rectal. Thyroid. Head and neck. Esophageal. Lymphoma. Lung. Pancreatic. Prostate. Melanoma.	Their use of elements of radioactive produce some damage, particularly to pregnant women. It is highly sensitive to diagnostic tools.	At early stages of neurological deceases, it is effective in diagnosis [41]. No risk of infection. The patient is exposed to less radiation. Precise. Reduces non-significant surgeries.

#### 3.1.3. Histopathology Images (HIs)

HIs have different biological structures and are based on pathologists’ knowledge in defining morphological and architectural features. Through the tissue area, HIs show an appearance with visual variability at a high rate in the linked patterns of certain small structures. In biological and anatomy systems, most of the visual variability is inherited [42]. For biological research, this useful modality, which makes it our main objective in this survey. The use of the HI modality affects the method of obtaining the design and data of algorithms concerning storage and processor limitations.

#### 3.1.4. Positron Emission Tomography (PET)

Mukai et al. [43] used 18F-fluoro-2-deoxy-d-glucose (FDG) to screen PET images for malignant tumors. They also evaluated preoperative PET images in cancer patients. The following sections present a detailed discussion of common imaging modalities.

### 3.2. Common Diagnosis Systems Based on HI Analysis

**Figure 7:**
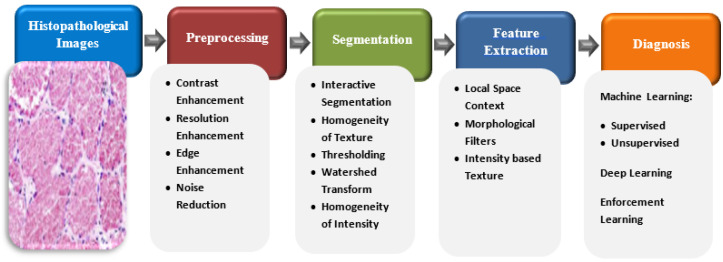
HI analysis pipeline.

In this paper, our main focus is on colon cancer diagnosis based on HI analysis. In general, most of the stages of HI analysis depend mainly on the basic concepts of mathematics. Figure 7 presents the main stages of a typical HI Analysis pipeline [44].

In the first stage, 2D/3D arrays of HIs are obtained and passed to a gray-scale or color imaging system. They are then fed to the preprocessing phase, where some operations in linear algebra are applied to array of the image for better image resolution to be able to distinguish structures from others. Then, the segmentation phase separates the background of the objects from the cells by applying mathematical algorithms, such as texture homogeneity, intensity, watershed transformation, and level set transformations.

The next stage is the extraction of features process. Instead of processing each pixel, this stage explores the most significant features from the sliced images for further processing. Therefore, it minimizes the computational complexity of the system. Finally, the diagnostic stage applies clustering or classification algorithms on the features extracted from the input images [45]. To achieve an intensive analysis of HIs, mathematical functions and operations must be applied to all analysis phases, beginning with the prepossession phase and ending with the diagnostic phase [46].

This section discusses common diagnosis systems applied for colon cancer detection based on HI analysis. These systems include Computer-Aided Diagnosis (CAD), Content-Based Image Retrieval (CBIR), and other findings in clinicopathology association systems.

#### 3.2.1. Computer-Aided Diagnosis (CAD) Systems

During the process of analysis of electronic HIs, CAD systems cover many of the tasks, and thus their functionality corresponds to the pathologists who are involved. The errors that result from applying the ML process differ from those that result from an individual pathologist. Therefore, the application of the CAD method improves the classification results and increases the system’s reliability. Furthermore, you can minimize the instability of analyzing and understanding each pixel in WSI. Various diagnostic functions involve the region of interest segmentation or recognition as an index of immunization.

Doi et al. [47] presented the motivation and strategy for the CAD scheme’s early development, together with the future potential and current status of CAD in an environment of PACs, using the output of the computer as a “second opinion” by radiologists with CAD to make the final decisions. The results were that the sensitivity was 75% at 1.03 false-positive fractures per image.

Hamilton et al. [48] presented, within large histological scenes, a means of locating abnormality focal areas through image texture scanning on low-power mode. In this study, images classified as dysplastic and normal on the basis of their texture were not perfect.

#### 3.2.2. Content-Based Image Retrieval (CBIR) Systems

Images used by CBIR are related to query images. CBIR approaches are supported by different histories, such as the study, training, examination, and pathology. For example, CBIR methods can be used by novice pathologists and academic applications to recover HIs from tissues appropriately. Furthermore, they are useful for competent pathologists, particularly when detecting unusual situations.

We can use unsupervised learning since CBIR does not require tagged data. There is a need for high-speed investigation from multiple images that depends on related images—not only accuracy—in CBIR. Therefore, the dimensional features of the image can be minimized by many approaches, such as nearest-neighbor searches, which are rapidly estimated [49].

Hou et al. [50] proposed supervised methods based on labeled histopathology data with large volumes that are expensive to generate. The proposed method learns in an unsupervised manner from heterogeneous pathology patches. The used model is synthesized with importance weights as trained patches, to train the task-specific (e.g., segmentation) CNN to minimize the ideal (unbiased) generalization error over real data.

In supervised methods, the results are significantly better than across-cancer generalization results when no supervised data is available for a cancer case. The proposed method does as well as supervised methods, even existing supervised data, due to the synthetic data being on a much larger scale. The results are segmented on over 5000 whole-slide images (WSIs), which is a larger dataset than the human annotated datasets that are currently available.

#### 3.2.3. Finding New Clinicopathological Association Systems

Analysts and pathologists produce many discoveries related to important diseases, such as contagious diseases and tumors. For example, pathologists processed the gastric mucosa of individuals that are diagnosed with gastritis. The morphological options of cancers must be linked. Tumor classification is important for various cancer cases, such as breast and prostate cancer, specifically in the treatment preparation and the patient process of diagnosis.

There is a significant growth in the digitization of clinical data, where it has boosted techniques to assess the genome. Therefore, today, we can achieve large amounts of electronic data, such as genome data, CT scans, and MRI. New relations of hospital pathology, such as somatic cancer mutations, including morphological quality, are available by examining the relationships between different imaging screening methods. Existing CAD approaches can be classified into DL and conventional ML methods, which will be discussed in the following section.

Cheng et al. [51] assessed mismatch repair protein deficiency in the largest series for the breast cancer as survival outcomes were linked and the immunohistochemistry was determined. They determined 31 MMR-deficient states out of 1635 that had data for all four MMR biomarkers (MSH2, MSH6, MLH1, and PMS2).

Kim et al. [52] proposed patients with PCNSL having the characteristics of low FDG uptake over a 10-year period. The data recommended that PCNSL was closely matched by the tumor to negative results, specifically with a low uptake for MUM1 expression. MUM1 plays an essential role in the differentiation, survival, and proliferation of cells.

Janowczyk et al. [53] showed a unified tool for the DP domain and the importance of DL according to its innate ability for learning useful features from data directly via seven use cases: the (a) segmentation of nuclei, (b) segmentation of epithelium, (c) detection of lymphocyte, (d) detection of mitosis, and (e) classification of lymphoma. They outlined a guide with insights for bridging the current knowledge gap between the DP domain and DL methods.

### 3.3. Datasets

Dramatically increasing the dataset size needed for testing training is a critical challenge [54,55]. There are public datasets in the electronic pathology course, including manual observations for HIs. These are helpful in the review process. Image artifacts (e.g., the zoom level and image resolution) and slide problems (e.g., smudges) have similarity ratios. However, all of these datasets are expected only in specific states of tumors, and there are several tasks that the existing databases do not handle. The publicly available datasets for colorectal cancer are summarized in Table 5 and are discussed below.

**Figure 8:**
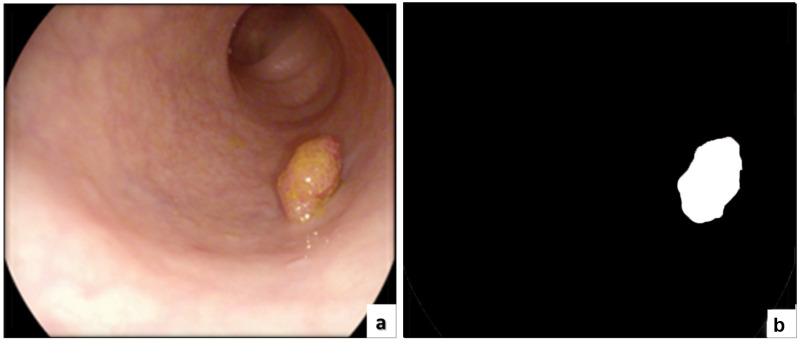
Original data and associated manual annotation from ETIS-Larib polyp DB. (**a**) the original image and (**b**) the annotation.

**Figure 9:**
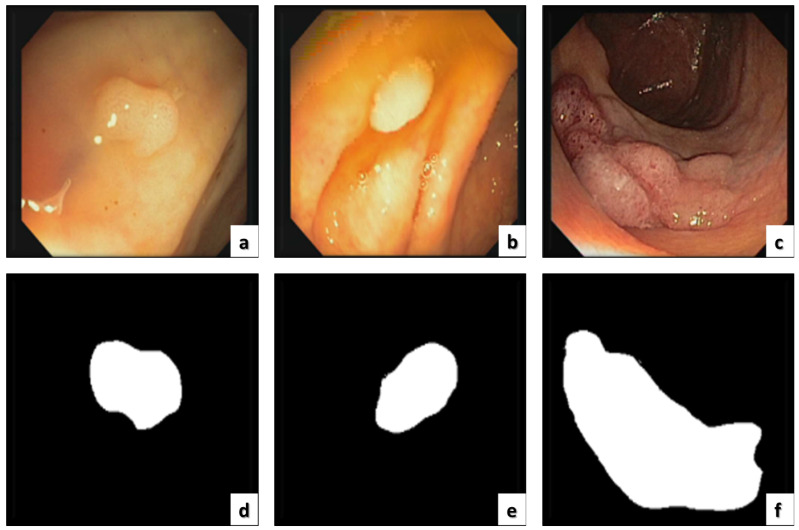
(**a**–**c**) The original images. (**d**–**f**) The corresponding ground truth.

1. **CRC Grading Dataset**
  The CRC [56] Grading Dataset contains 38 H&E stained histological WSIs with a resolution 4548 × 7548.
2. **PanNuke Dataset**
  PanNuke [57] includes 200,000 nuclei divided into five main classes to challenge the approaches of classifying and segmenting nuclei in WSIs with a resolution of 224 × 224.
3. **The Warwick-QU Dataset**
  In this dataset [58] are 16 slides of H&E stained histological WSIs of colon histology; this dataset is being created as category of the GlaS challenge with resolutions of 430 × 575 (14 images) and 520 × 775 (151 images).
4. **CoNSeP Dataset**
  CoNSeP [59] contains 41 H&E stained image slides with a resolution of 1000 × 1000 pixels at 40× magnification of objective: generally 24,319 annotated nuclei with labeled classes.
5. **ETIS-LARIB**
  The ETIS-LARIB [60] database contains frames taken from colonoscopy videos, including several examples of polyps. It produces the baseline reality for each frame while displaying a mask due to the polyp region in the image. A sample of this dataset is shown in Figure 8.
6. **CRCHistoPhenotypes–Labeled Cell Nuclei Dataset**
  This dataset [61] has 100 H&E CRC. For the process of detection, there are 29,756 nuclei; for classification, 22,444 nuclei (miscellaneous, fibroblast, and epithelial); and 7312 unlabeled with a resolution of 500 × 500.
7. **Kent Integrated Dataset (KID)**
  The KID [62] is responsible for the health and welfare system for the entire population of Medway and Kent. This dataset is rich and unique for researchers seeking health and care on a large scale. This also provides an overview of the patient journey, care, and needs.
8. **CVC-ColonDB and CVC-ClinicDB**
  Since 2012 [60], this dataset has been the top research leader as it includes many databases that are public and available, and CVC-ColonDB is included, which specializes in colon cancer imaging containing the original images and the ground truth as shown in Figure 9.
9. **Colonoscopy Dataset**
  The dataset [63] contains 76 videos, containing both WL and NBI. The database contains 40 adenomas with SD resolution of 768 × 576, 21 hyperplastic lesions, and 15 serrated adenomas.
10. **Extended CRC Grading Dataset (KID)** In this dataset [64] are 300 images that are non-overlapping. These were labeled by expert pathologists as high grade (Grade 3) tumors, low grade (Grade 2) tumors, or normal tissue (Grade 1) with a resolution of 4548 × 7548.
11. **ASU-Mayo Clinic**
  Currently, there are numerous research programs based on co-funded acceleration, seed research, and team science grants [65]. This means that more than 20–30 cohorts of senior nursing students in their clinical training by Mayo Clinic nursing faculty on the Mayo campus are expected to be completed. Due to this effort and cooperation, the seed grant program has added joint, cutting-edge research collaborations, a host of dual degree opportunities, and others. In 2016 and in the summer of 2010, the relationships of the Mayo Clinic became enterprise-wide, and the ASU Alliance for Health Care was formed.

**Table 5:** Datasets for colorectal cancer with different imaging modalities.

Author	Dataset Name	No. of Images	Resolution	Task	Modality
Awan et al. [56]	CRC Grading Dataset	139	4548 × 7548	Classification, Cancer grading,	Histology
Gamper et al. [57]	PanNuke Dataset	20K WSI	224 × 224	Classification, Segmentation	Histology
Sirinukunwattana et al. [58]	The Warwick-QU Dataset	165	430 × 575 (14 images), 520 × 775 (151 images),	Gland segmentation	Histology
Leenhardt et al. [66]	CAD-CAP	25,000	Various resolutions	Detection, classification	Capsule endoscopy (CE)
Graham et al. [59]	CoNSeP dataset	41	1000 × 1000	Classification, Nuclear instance segmentation	Histology
Bernal et al. [67]	ETIS-Laribv	196	HD,1225 × 966	Polyp detection, localization	Colonoscopy
Vázquez et al. [68]	CVC-ColonDB	300	SD, 574 × 500	Polyp segmentation, localization	Colonoscopy
Sirinukunwattana et al. [61]	CRCHistoPhenotypes–Labeled Cell Nuclei Dataset	100	500 × 500	Nucleus detection, classification	Histology
Bernal et al. [69]	CVC-ClinicDB	612	SD, 384 ×b288	Polyp detection, localization, segmentation	Colonoscopy
Jha et al. [60]	Kvasir-SEG	1000	Various resolutions	Polyp segmentation	Colonoscopy
Mesejo et al. [63]	Colonoscopy Dataset	76	SD, 768 × 576	Classification	Colonoscopy
Javed et al. [70]	CRC Tissue Phenotyping (CRC-TP) Dataset	256	500 × 500	Nucleus detection, classification	Histology
Kather et al. [71]	CRC-VAL-HE-7K CRC-VAL-HE-7K	7180 image patches	224 × 224	Predicting survival, classification, detection	Histology
Shaban et al. [64]	Extended CRC Grading Dataset	300	4548 × 7548	Cancer Grading	Histology

### 3.4. Performance Evaluation Metrics

Metrics of evaluation are utilized to measure the quality of models of machine learning. One can evaluate whether the DL algorithm of training is effective on new data by using these metrics of evaluation. Many different evaluation metrics can be used for testing a model. More accurate results can be found using multiple metrics for evaluating the quality of a trained model because each model performing using a metric of evaluation differs from the same model using another evaluation metric.

The factors of correctly used evaluation metrics are critical as these describe whether the trained model is performing well or not. In the following section, we show some formulas and an explanation of the evaluation metrics utilized by academic papers.

True Positive (TP) is when a method classifies the correct category correctly, while False Positive (FP) is when a method classifies the correct category incorrectly. On the other hand, True Negative (TN) is when a method classifies the negative category correctly, while False Negative (FN) is when a method classifies the negative category incorrectly. We can customize these values in the medical field of cancer detection. An example is that, if the image includes cancerous cells, then the trained model predicts the malignant cells successfully, and thus this case is called TP, while if the trained model predicts that it is not a malignant cell, then this case is called FP.

On the other hand, if the image includes no malignant cells, and the model predicts that the image does not contain cancerous cells, then this case is called TN. If the image includes no malignant cells, and the trained model predicts it as a malignant cell, then this case is called FN. In the next section, we present an explanation and description for formulas that are related to the common evaluation metrics.

- **The Accuracy** measures the proportion of true observations to the number of samples measured, which can be calculated as:
  $$\mathrm{Accuracy}=\frac{\mathrm{TP}\;+\;\mathrm{TN}}{\mathrm{TP}\;+\;\mathrm{TN}\;+\;\mathrm{FN}\;+\;\mathrm{FP}}$$
- **The Rate of Error** shows the proportion of inaccurate observations to the number of measured samples, which can be calculated as:
  $$\mathrm{Error}\;\mathrm{Rate}=\frac{\mathrm{FP}\;+\;\mathrm{FN}}{\mathrm{TP}\;+\;\mathrm{TN}\;+\;\mathrm{FN}\;+\;\mathrm{FP}}$$
- **The Precision** measures the true classified positive estimates of the total classified estimates in a correct category, which can be calculated as:
  $$\mathrm{Precision}=\frac{\mathrm{TP}}{\mathrm{TP}\;+\;\mathrm{FP}}$$
- **The Recall** is employed for measuring the ratio of correct estimates that are correctly predicted. This can be calculated as:
  $$\mathrm{Recall}=\frac{\mathrm{TP}}{\mathrm{TP}\;+\;\mathrm{FN}}$$
- **The Specificity** is presented for measuring the positive observations rate of false samples and can be calculated as:
  $$\mathrm{Specificity}=\frac{\mathrm{TN}}{\mathrm{TN}\;+\;\mathrm{FN}}$$
- **The Sensitivity** measures the number of correct samples that are classified as true and can be calculated as:
  $$\mathrm{Sensitivity}=\frac{\mathrm{TN}}{\mathrm{TN}\;+\;\mathrm{FN}}$$
- **The ROC curve** presents the ratio of false positives to the ratio of TPs by showing the performances of the possible threshold values used and can be calculated as:
  $$\mathrm{TPR}=\frac{\mathrm{TP}}{\mathrm{TP}\;+\;\mathrm{FN}}$$
  $$\mathrm{FPR}=\frac{\mathrm{FP}}{\mathrm{FP}\;+\;\mathrm{TN}}$$

## 4. Literature Review

CAD systems are an active topic to research using HIs and play an important role in diagnosis. Various imaging techniques are used to diagnose the disease and examine these HIs. In addition, various MIA techniques have been performed for classification to measure disease characteristics from HIs. Additionally, nuclei and glands can be segmented to recognize cell types and to automatically determine the existence of a disease within samples. Depending on the sample, the evaluation of the intensity of the disease can also be obtained [72].

Existing CAD systems can be categorized into systems based on ML techniques and systems based on DL techniques. In the following subsections, we introduce the most prominent research proposed toward the diagnosis of colon cancer on the basis of ML and DL techniques.

### 4.1. Conventional Machine-Learning Methods

**Figure 10:**
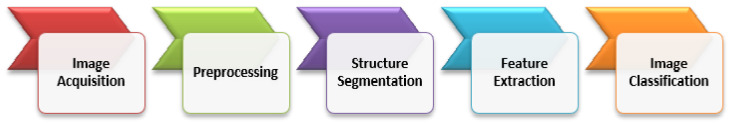
The main stages of conventional ML methods for HI analysis.

The machine-learning process for HI analysis involves five main phases as shown in Figure 10, and these are discussed in the following text.

#### 4.1.1. Preprocessing

The images obtained contain variations (e.g., staining, color, and noise) and must be uniform. These variations generally result from the scanning procedures. The components and architecture of the tissue are analyzed using wax under the microscope to produce the macroscopic sections. Pathologists use one or more colored stains to diagnose and analyze the architecture and tissue components to isolate the cellular components [73].

Jang et al. [74] used hematoxylin-eosin (H&E) to separate the nuclei, cytoplasm, and connective polyps. Hematoxylin colors the nucleus blue and eosin colors the cytoplasm and connective tissue pink. The classification performance is determined based on the consistency of the extracted features. Therefore, the step of image processing is essential for determining the image state. On this basis, the approaches utilized to enhance the image and to overcome different illumination fluctuations are determined. In this way, the quality is also improved.

Various preprocessing approaches can be applied to images. The preprocessing techniques are well adapted to the state of the image. They control the differences of contrast in images, noise removal, and brightness. Therefore, this phase is critical as the input tissue images must be similar to those stored in the database. In addition, a number of effects, such as image size variations, orientation, posture, lighting, and background, should be reduced.

#### 4.1.2. Segmentation

The process of segmentation in images plays an essential role in the analysis of histopathology images, which plays a significant role in solving different problems. The tasks required are different from each other for each stage, and even each image is different from another. Image segmentation is similar to clustering. This defines meaningful segments that can differ from model to model or even cell to cell [28,75].

#### 4.1.3. Feature Extraction

This stage is utilized for extracting features from the colonoscopy images that describe the characteristics of the colon. This is performed using computer-diagnosed colonoscopy images to discover the patient’s current condition. At this stage, tissues are removed from the region to facilitate the diagnostic process [76]. This step helps to improve the accuracy of the classification performance. There are many feature extraction techniques, such as LBP [77], SIFT [78], and HOG [79].

There are many available approaches for examining the colon, such as barium radiography and sigmoidoscopy; however, colonoscopy is currently the best modality for diagnosing any colonic tissue. The early detection of any tissue increases the chances of a cure for patients.

Image textures in CTCs have a high capability to differ between various tissue cases and thus improve the CTC model toward optimal tissue management for preventing fatal colorectal cancer [80]. However, textures of images are frequently compromised according to the operations of error correction and noise smoothing in most CT image reconstructions.

#### 4.1.4. Image Classification

Accurate and efficient histological cell classification is paramount for image analysis in the medical field. Thus, the classification process is a challenging task according to the variation of the cells. For doctors, this phase makes it easier to understand the different treatment approaches for colon cancer. Below, we review recent ML-based studies for colon cancer diagnosis, and these are summarized in Table 6. After a comprehensive analysis of these studies, we summarize their main strengths and limitations in Table 7.

Niazi et al. [81] showed the use of unsupervised learning for colon cancer detection and the process of cancer case analysis using gene expression data. This method differs from any other approach used for diagnosing colon cancer, as it allows the use of various kinds of data and is not only for colon cancer. Gene expression data are used to classify and detect different types of cancer.

Rasti et al. [82] proposed three approaches of classification to three states of cancer on mice colon walls as well as inflammation and health with the use of endomicroscopy images. Fully automated methods of machine learning (ML) were presented with the SVM methodology, including classical texture-based classification, transfer learning, and deep learning. They compared various strategies of training. The experimental results had an accuracy of 99.93% on the ImageNet/ILSVRC dataset.

Na et al. [83,84,85] presented a fully automated method of classification with a GBM method that depends on supervised learning, which was then tested on many images. The experimental results showed that the rate of correct recognition was 99.93%, which was the best performance proved for the second approach. For the more difficult first case, the results were 98.49%. They utilized the CRCHistoPhenotypes dataset, which includes a large number of microscopy images.

Rathore et al. [86] presented a complete approach for gland segmentation and for cancer classification and detection into three different colon cancer grades. For the segmentation of colon glands, they modeled the tissue parts as ellipsoids. For the process of detection and classification, they extracted multiscale features that encode the texture and the spatial architectural patterns of a gland apart from its cellular morphology. They evaluated their study using two different datasets for the classification into three cancer grades.

Dragicevic et al. [87] presented a colon detection model on the basis of a new imaging method called Optomagnetic Imaging Spectroscopy (OMIS). In comparison with a histopathological imaging model, the OMIS showed results with an accuracy of 92.59% using MPNN as a classifier and 89.87% using Naïve Bayes.

Shanmuga et al. [88] showed a CAD system that utilizes the WCE method for screening to determine tumors of colon cancer. In the proposed method, the images are entered to the preprocessing stage using filtering and ROI-based histograms. Then, the tumor region is detected using an algorithm of K-means clustering. From the segmented regions, features are extracted using spatial gray level dependence matrices (SGLDM). Finally, a support vector machine is applied for tumor multilevel classification into benign, malignant, or normal. This detection method achieved an accuracy of 95%.

Babu et al. [89] proposed a multi-level threshold image segmentation approach based on 2DReCA. In addition, they extracted two sets of textures from the preprocessed grayscale colon images. They evaluated the quality of the presented model using a random forest classifier on different images data of colon cell sets with different magnification factors containing malignant and normal labels.

Fahami et al. [90] employed methods of machine learning, such as KNNs and DT, to determine the properties of tumors of this cancer. The main task in the process of colon cancer diagnosis using HIs is the prediction of cancerous genes. To this end, various algorithms of ML have been utilized on the data of colon cancer. This research utilized supervised and unsupervised ML methods to predict the top active genes in patients of colon cancer. They categorized the patients into two main sections and explored the most effective 20 genes of the vital status in each section. The experimental results had an accuracy of 97.49 ± 2.92 on the HTSeq-FPKM-U dataset.

Jansen-Winkeln et al. [91] proposed an approach of colon cancer detection using HSI optical imaging technology. They utilized a four-layer perceptron neural network to classify the image into three classes: adenomatous margin close to the central tumor, cancer, and healthy mucosa. They evaluated their work using images collected from patients between July 2019 and May 2020 using a hyperspectral camera. Their results reached a sensitivity of 86%.

Talukder et al. [92] showed a combination of deep-leaning and machine-learning techniques for the detection of colon and lung cancers. They combined the deep features extracted using deep-learning techniques with ensemble learning machine-learning classifiers. They evaluated their model using the LC25000 dataset for lung and colon cancers. Experiments were performed for colon and lung cancer with accuracy ratios of 100% and 99.30%, respectively.

Chehade et al. [93] presented a CAD system that classifies HIs of colon and lung cancer into five different classes. In this model, HIs were preprocessed using image preprocessing techniques, and then the discriminative features were extracted. Finally, they fed the extracted features into six machine-leaning models: MLP, SVM, LDA, XGBoost, RF, and LightGBM.

Alqudah et al. [94] presented an ML-based model for the detection of colon cancer. They utilized 3D GLCM matrices of three different color spaces to extract texture features from the input HIs. Then, the extracted features were fed into five different ML algorithms: SVM, ANN, KNN, QDA, and CDT. This model was evaluated on a private dataset containing eight different classes of HIs: tumors, debris, lymphoma, adipose, complex, empty, mucosa, and stroma.

### 4.2. Deep-Learning Methods

DL methods have successfully produced many models for processing images, [95] and for processing voice/sound [96]. Recently, researchers presented that the DL can also be utilized in the processing of medical images, such as MRI [97], CT [98], biopsy [99], and endoscopy [100] images. Generally, datasets of digital pathology have become increasingly available and public. It has became possible and feasible to evaluate DL techniques to enhance the quality and efficiency of histological diagnosis.

Recently, DL techniques have been used in various fields and have achieved superior results compared to traditional ML methods (for example, automated analysis for HIs, natural language processing (NLP), and biomedical fields). With DL, abstract representations are presented in a meaningful way and can be quickly understood. CNN is considered a typical instance of applied structure. When using techniques of DL, several measures can be utilized to manage histopathology depending on the task settings.

**Figure 11:**
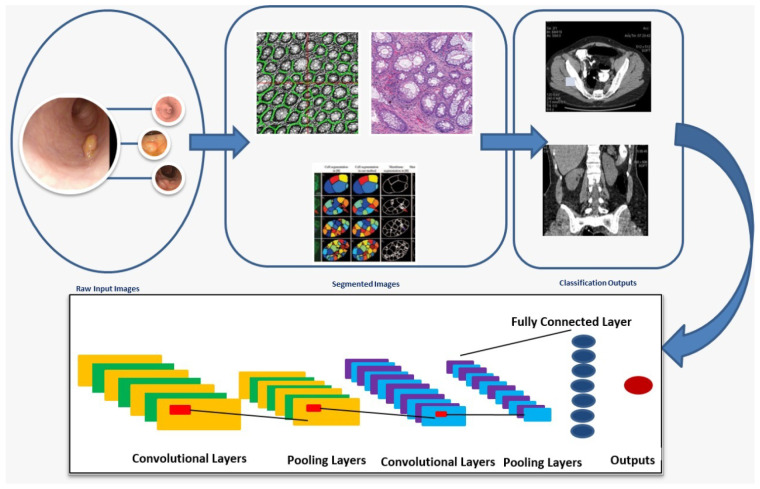
The segmentation and classification process using deep learning.

HIs styles consider the primary features that the scaling stages can determine. There are two primary factors: patch size, which is network related, and impure homogeneity for whole-slide images (WSI) [48]. The network structure shows the main position. However, predefined system structures are maintained in many studies. Figure 11 presents the main steps for the segmentation and classification of colon cancer using a DL-based Convolutional Neural Network (CNN) architecture.

The following paragraphs will review the common DL-based research for colon cancer diagnosis. A summary of these studies is presented in Table 8, and a comparison identifying their main strengths and limitations is presented in Table 9.

Shapcott et al. [101] used a cell-identification DL algorithm in TCGA for colon cancer imaging, which improved the performance without loss of accuracy when sampling the image [102]. The extracted features were related to various variables, including: venous invasion, lymphatic invasion, metastasis, and residual tumors. The local dataset was used to train the DL algorithm, and then TCGA images were used for testing. In each part, they identified the tissues. The average number of slides in an image containing cells in this application was 900.

De et al. [103] showed a method for automatic polyp insertion detection in colonoscopy images. They utilized ETIS-LaribPolypDB as a testing set and the CVC-ClinicDB database as a training set. The results of their method showed that the process of polyp insertion is useful to reduce false positive (FP) and that the traditional augmentation of data can be effective. The results showed a low false positive rate (FPR) while maintaining a substantial sensitivity/recall. The final results showed that the F1-Score was 91.4% and the FPR was 0.079 with their modified version of a training set over the ETIS-LaribPolypDB testing dataset.

Kang et al. [104] presented an ensemble transfer-learning model based on the union of two classified masks by bitwise operations for the segmentation of colorectal polyps. They used the CVC-ClinicDB, CVC-ColonDB, and ETIS-Larib datasets. The results of their experiments demonstrated the superiority of their approach against state-of-the-art approaches to segmenting polyps.

Sornapudi et al. [105] presented a modified R-CNN by creating masks around the detection of tissues from frames. This approach was developed on the basis of the R-CNN. The generated feature maps utilized ResNet-101 and produced further details using FPN for polyp images compared to ResNet-50. The proposed model can be segmented and detected the polyps in images successfully. This approach can preform a segmentation process accurately for each polyp. This produced better quality on the WCE video frames compared with images of colonoscopy. The results showed better polyp localization compared with recent DL and traditional methods.

Zhang et al. [106] presented SSD-GPNet, which is an improved SSD for gastric polyp detection in real-time with 50 FPS using Titan, which is a CNN that is produced based on SSD architecture. They utilized images of colonoscopy containing two independent datasets and a special dataset consisting of 2484 images. The experimental results presented that the improved SSD for the detection of gastric polyps can be applied for real-time polyp detection with 50 FPS and can enhance the mAP from 88.5% to 90.4% with low performance times.

Zobel et al. [107] applied an R-CNN. They utilized three colonoscopy independent datasets, including 2484 HD labeled tissue images from their clinic, as well as two public datasets from the MICCAI 2015 detection challenge for polyps, containing 194 HD labeled images and 612 SD with polyps. The experimental results showed that the three datasets were investigated with precision = 0.86, recall = 0.92, F1 = 0.89 (dataset A), precision = 0.80, recall = 0.86, F1 = 0.82 (dataset B) and precision = 0.74, recall = 0.83, and F1 = 0.79 (dataset C).

Ma et al. [108] proposed a DL model using a CNN for efficiently classifying and detecting colorectal polyps from images of colonoscopy. They trained their model using a dataset that is a benchmark and showed results of 92% accuracy and efficient computational speed for all sizes of polyps, which might be overlooked in colonoscopy. Blanes-Vidal et al. [109] introduced an algorithm to objectively quantify the similarity between predicted polyps and those classified by OC, CCE, and HP, depending on their morphology, location, and size. They also proposed a CNN for the autonomous localization and detection of colorectal polyps in WCE. They used WCE images from datasets, and the results produced unexpectedly high sensitivity (97.1%), accuracy (96.4%), and specificity (93.3%).

Wang et al. [110] showed a CAD real-time system based on DL directed to maximize the detection rates of adenoma in a low-prevalent ADR region in colorectal tissue. Given fidelity, stability and high accuracy, their system can be applied in clinical practice for better quality detection of colon polyps.

Yuan et al. [111] produced a model of DenseNet-UDCS to detect tissues from the WCE images, considering small inter-class variances in the dataset, the image unbalanced problem, and large intra-class differences. Their results found a tissue recognition accuracy of 93.19%, presenting that the proposed DenseNet-UDCS could detect polyps from the images and could classify the endoscopic images accurately.

Jia et al. [112] proposed PLPNet, which is a two-stage framework for the detection of pixel-accurate tissues in images of colonoscopy that achieved accuracy using a CNN. They utilized the MICCAI 2015 dataset with results that improved on the generality and effectiveness of the produced system. PLPNet is effective, simple, and fast at inference and can be suggested for applications in clinical practice.

Tripathi et al. [113] presented a method using histopathological images that combined deep and handcrafted features utilizing medical datasets (ImageNet). Two datasets of colon cancer nuclei were used. They combined methods of DL in different ways, which led to the best approach that used AlexNet, VGG16, VGG19, ResNet50, DenseNet121, and InceptionV3 methods. Then, they combined handcrafted features that directly depended on raw images instead of object-level features.

For better performance, they used weak descriptor features to remove the artifacts present around the nuclei and background. To reduce space and computational complexity, they combined techniques of handcrafted and deep networks. They utilized an experimental dataset by randomly categorizing the dataset into testing, validation, and training subsets of 15%, 70%, and 15% respectively, and the network was enhanced by 15% on the validation sets and 70% on the training set.

Javed et al. [70] showed a detection algorithm for a cellular community with semi-supervised phenotyping of tissue. This depended on polyp classification, clustering, and the detection of image patches into communities with meaning. For cell classification and detection, they first used deep neural networks and then utilized tissue–tissue links between these tissues for computing feature vectors at the slide level. For constructing a network at patch level, they used these feature vectors through the chi-square distance, where the weights of each node and edge were inversely proportional to the distance between the vectors of the feature.

They proved that their algorithm is for both handcrafted and deep-learning features that complement each other. The results were: specificity 0.920%, accuracy 0.898%, recall 0.898%, precision 0.936%, and F1 0.914% for the Etis-Larib dataset. The results for the CVC-ClinicDB dataset were: specificity 0.994%, accuracy 0.985%, precision 0.985%, F1 0.985%, and recall 0.985%.

Shaban et al. [64] presented a context-aware CNN for cancer staging capable of a context that was 64-times larger than normal CNN-based slide classifiers. The quantitative and qualitative results showed that their method enhanced domain-oriented techniques, methodologies of classification based on patches, and existing methods of context.

Nadimi et al. [114] presented a CNN for autonomous colorectal tissue detection in images captured during WCE in the evolution of colorectal cancer with the risk of malignancy. They utilized Kvasir datasets and WCE images from independent datasets, and the results showed an unprecedented sensitivity of 98.1%, a high accuracy of 98.0%, and a specificity of 96.3%.

Mostafiz et al. [115] presented a powerful detection system of gastrointestinal polyps in endoscopic videos. The presented method explains an automatic system depending on an extraction of new schemes of colored features as support for the gastrointestinal detection of polyps. Their system showed higher accuracy from the analysis of ROC. The experimental results on standard public databases produced that the presented system outperformed the previous conventional approaches, obtaining a sensitivity of 99.91%, accuracy of 99.53%, and specificity of 99.15%.

Ozawa et al. [116] used a Single-Shot Multi-Box Detector, which is a CNN. They utilized 4013 images of normal colorectums and 16,418 images from 4752 CPs for training, and then using 7077 colonoscopy images, considering 1172 CP images from 309 different kinds of CP for validating. The speed of diagnostics for the classification and detection of CP were measured as a factor of quality of the trained model. The trained network detected 1246 CP with a PPV of 86% and a sensitivity of 92%. The PPV and sensitivity were 83% and 90%, respectively, for the white light images, and 98% and 97% for the narrowband images.

**Table 8:** Summary of DL-based studies for colorectal cancer diagnosis.

Author (Year)	Topic	Imaging Modality	DL Architecture	Datasets Availability	Results
**Hou et al. [50] (2019)**	Polyp segmentation	HIs	CNN	Public	Reduce the error of segmentation by 7.8%, 5.4%, and 3.2%.
**Janowczyk et al. [53] (2016)**	Polyp Detection	Digital-Pathology (DP)	NIA	Public	TPR: 86% PPV: 64%
**Tripathi et al. [113] (2020)**	Polyp Detection	HIs	AlexNet VGG16 VGG19 ResNet50 DenseNet121 InceptionV3	Public	Precision: 0.62% Recall: 0.63% AUC: 0.03% Loss: 0.0043
**Shapcott et al. [101] (2019)**	Polyp detection	HIs	CNNs	Private	Accuracy: 65%
**Ben Hamida et al. [117] (2021)**	Polyp detection	Digital pathology (DP)	ALEXNET	Public	CRC-5000-Accuracy: 98.66% NCT-CRC-HE-Accuracy: 99.12%
**Liewa et al. [118] (2021)**	Polyp Detection CVC-ClinicDB	Endoscopic Images	ResNet-50	Public	Accuracy: 99.10% Sensitivity: 98.82% Precision: 99.37% Specificity: 99.38%
**Pacal et al. [20] (2020)**	Polyp Detection	CT	RNNs, Autoencoders (AEs)	Public	sensitivity: 0.91
**De et al. [103] (2019)**	Polyp Segmentation	Colonoscopy Images	CNNs	Public	F1-Score: 91.4% FPR: 0.079
**Javed et al. [70] (2020)**	Detection of Polyp	Colonoscopy Images	CNN	Public	Specificity: 920% Accuracy: 89.8% F1: 91.4% Recall: 89.8% Precision: 93.6%
**Sikder et al. [119] (2021)**	Polyp Detection	MRI	CNN	Private	Accuracy: 93%
**Kang et al. [104] (2019)**	Polyp Segmentation	Colonoscopy	CNN	Public	Dataset results of Etis-Larib: recall: 74.37%, precision: 73.84%, IoU: 66.07%
**Sornapudi et al. [105] (2019)**	Detection of Polyp	WCE + Colonoscopy	CNN	Public	Dataset results of ResNet-101: F2: 78.70% recall: 80.29%, F1: 76.43%, precision: 72.93%. Dataset results of ResNet-50: recall: 67.79%, F2: 66.57%, precision: 62.11%, F1: 64.83%
**Jia et al. [112] (2020)**	Segmentation of Polyp	Colonoscopy	CNN	Public	recall: 81.7%, Precision: 63.9%, F2: 77.4%, F1: 71.7%
**Ozawa et al. [116] (2020)**	Polyp classification of colorectal and automated detection of endoscopic	Colonoscopy	CNN	Private	Detection: PPV: 86%, sensitivity: 92% Narrow-band images classification: 81% Conventional white-light Classification images: 83%
**Zhang et al. [106] (2019)**	Detection of Polyps	Colonoscopy	CNN	Private	F1: 84.24%, recall: 76.37%, Precision: 93.92%
**Zobel et al. [107] (2019)**	Polyp Detection	Colonoscopy	CNN	Private	F1: 89%, precision: 86%, Recall: 93%
**Ma et al. [108] (2019)**	Polyp Localization	Colonoscopy	CNN	Private	sensitivity: 93.67%, accuracy: 96%, AP: 94.92%, specificity: 98.36%
**Shaban et al. [64] (2020)**	Polyp Detection and Classification	Colonoscopy	CNN	Private	F2: 66.07%, F1: 68.72%
**Blanes-Vidal et al. [109] (2019)**	Polyp detection	WCE	CNN	Private	sensitivity: 97.1%, Accuracy: 96.4%, specificity: 93.3%
**Wang et al. [110] (2019)**	Real-time automatic detection system	Colonoscopy	CNN	Private	ADR Increment 9.1% vs. 20.3%, *p* < 0.001)
**Mostafiz et al. [115] (2020)**	Polyp Detection	Colonoscopy	CNN + CEMD	Public	sensitivity: 99.91%, Accuracy: 99.53%, specificity: 99.15%
**Yuan et al. [111] (2019)**	Polyp Recognition	WCE	CNN	Private	Accuracy: 0.9319, precision: 74.51%, recall: 90.21%, F1: 81.83%
**Nadimi et al. [114] (2020)**	Colorectal polyps Localization and Autonomous Detection	WCE	CNN	Private	sensitivity: 98.1%, Accuracy: 98%, specificity: 96.3%
**Javed et al. [70] (2020)**	Detection of Cellular Community for Issue Phenotyping	Histopathology	Handcrafted, CNN	Public	Patch level separation: average F-score for CCT dataset: 94.5%, average F-score for CRC-TP dataset: 91% Patient level separation: average F-score for CRC-TP dataset: 84%

**Table 9:** General strengths and limitations of DL studies.

Author	Strengths	Limitations
**Hou et al. [50] (2019)**	Their approach generalizes significantly better to cancer cases without training data. Investigated the best quality without supervision cost.	Not generalized with mixed-quality image classification.
**Shapcott et al. [101] (2019)**	Reported cellularity, which has been linked to patient and prognostic indicators and other diagnostic.	Not handling small clusters or tumor polyps of up to five polyps in the stroma, which is associated with aggressive cancer.
**Ben Hamida et al. [117] (2021)**	Their method improved the results when treating with a sparsely annotated dataset	Techniques for accuracy not compatible with computational cost balance.
**Liewa et al. [118] (2021)**	Their approaches are robust enough to assist in CAD	Their classification system can misclassify images taken by colonoscopy/endoscopy according to the structure and image color characteristics, which are naturally irregular in the colon.
**Pacal et al. [20] (2020)**	Presented a comprehensive survey with all overviews.	Their model did not determine a common experimental setup and evaluation criteria.
**Sikder et al. [119] (2021)**	The method is pointedly precise, supported, and practical. Can detect malignant cells automatically. Their collection gives high accuracy, particularly after performing the algorithm of ML.	For large datasets, the used algorithm showed high complexity time. Low accuracy rate.
**Kang et al. [104] (2019)**	Used a strong object for detection CNN called Mask R-CNN. Utilized a successful ensemble model for combining the two masked approaches of R-CNNs with various backbone structures.	Less backbone structures. Less efficient segmentation, However, the successful ensemble method should be used with backbone structures.
**Sornapudi et al. [105] (2019)**	Successful detection and accurately used the segmentation method. The proposed approach showed better performance on the WCE video frames than images of colonoscopy.	Used the Etis-Larib dataset that does not produce efficient precision Training data is not sufficient for an accurate model.
**Jia et al., [112] (2020)**	Improved the residual learning and the feature pyramids. Developed the segmentation task of polyps.	Less stage integrations of PLPNet.
**Zobel et al. [107] (2019)**	Reduced the computation time. Detected too many FB areas.	The small training database for training a Mask R-CNN with a ResNet-101 backbone.
**Ma et al. [108] (2019)**	Overcome the problems of overfitting and gradient vanishing.	Not enough images for a training model.
**Shaban et al. [64] (2020)**	Well-suited for the CRC staging task.	Not efficient for digital images at the whole-patch level for the analysis of patient survival.
**Blanes-Vidal et al. [109] (2019)**	The used algorithm was able to determine the polyps’ similarity and determine the degree that is related to how true a match is. The used algorithm can be generalized.	High cost of information required on the location assessment and the polyp morphology. The detection algorithm was not efficient with the number of used images.
**Wang et al. [120] (2020)**	Achieved the effect of an automatic detection system for polyps, which was dependent on the DL for the detection rate for polyps and ADR.	The proposed system may be difficult to evaluate. Lack of external validity. False-positives rates were low. Fatigue level of participating endoscopies were not controlled for in this system, which considered this as an independent factor on ADR.
**Mostafiz et al. [115] (2020)**	Produce computer-aided system with great accuracy.	Small amount of FP and FN values.
**Yuan et al. [111] (2019)**	Model of DenseNet-UDCS was superior in accuracy of detection.	In the dataset, there are variances of small inter-class and unbalanced images and large intra-class differences.
**Nadimi et al. [114] (2020)**	The general rules are task-independent with less ambiguity for optimal feature selection. Better results compared with other state-of-the-art detection of polyps by a wide margin. Network predictions are given more interpretability.	Did not produce sufficient concrete interpretability.
**Ozawa et al. [116] (2020)**	Trained CNN presented a robust result for the detection and classification of CP.	This is retrospective research in a single association.

Ben Hamida et al. [117] proposed and assessed state-of-the-art models of DL for the pixel- and patch-level classification of a sparsely annotated dataset for colorectal histopathology. They presented, from a dataset of generalized multimedia, the utilization of transfer learning to a specific context of histopathological images. They used available datasets, such as the AiCOLO, ImageNet, and the GlaS datasets, but still had certain limitations, including gradient-vanishing problems, the main weakness that classical CNNs suffer from, which can control their ability to provide representations of generic data. Consequently, various improved techniques were produced, called RNN and inception models [28,37,38].

Liewa et al. [118] showed a new aggregation of a PCA with modified deep-residual CNNs and an ensemble-learning model for a colonic classification system of polyp. They evaluated their model by computing the MCC, sensitivity, accuracy, specificity, and precision. In the experiment of 1517 images from a collection of three free databases that are public and accessible, they acquired beneficial results with 0.9819 MCC. The precision, specificity, accuracy, and sensitivity of tissue classification were 99.37%, 99.38%, 99.10%, and 98.82%, respectively. Therefore, these results produced approaches robust enough to help in CAD. They used some available datasets, including Kvasir, CVC-ClinicDB, and ETIS-LaribPolypDB.

## 5. Current Challenges

This survey provided an overview of colon cancer and its stages to be diagnosed to determine the appropriate treatment. ML and DL techniques are used in image-processing methods to accurately detect this type of cancer. These methods play an essential role in many applications, such as construction, image recognition, health assessments, medical diagnosis, and defect identification. However, there exist many challenges facing the colon cancer diagnosis process. In this section, we discuss the challenges related to the process of colon cancer diagnosis, which are classified into two main aspects as introduced below.

### 5.1. Challenges Related to Image Modalities

This survey discusses the different modalities used to image tumors. MIA is an active topic to research in the ML area. Colorectal cancer screening uses a common method, which is HIs, where images have been taken using microscopes to locate, examine, classify, and provide a comprehensive view of the grades of cancer. Each modality has its strengths. By merging images from multiple modalities, we combine their strengths; however, this also has its flaws as discussed in this survey. Below, we list the most common challenges related to different imaging modalities.

- **CTC Images:**
  These have several challenges when isolating and enumerating. The first is that the cells have various structures, which creates various results for each tool in the image’s manual review. Thus, we demand a consistent and clear explanation of classification of the CTC [121].
- **MRI Images:**
  MRI has a challenge, which is the discomfort for patients while capturing these images, particularly in the anal stenosis case as well as to the movement of the rectal wall, which also has effects on these images. In routine cancer grading, the use of phased array coils is recommended to handle these motifs because the pelvic coil has been shown to produce high accuracy rates of 59–95% in rectal cancer [122,123].
- **PET Images:**
  The challenge with PET is that it only works by absorbing glucose. Although glucose is widespread in most body cells and can be absorbed and predicted, unlike malignant cells, it is difficult to detect in certain other tissues using the PET modality [124].
  The use of a combination of CT with PET produces better results, particularly in oncology, to diagnose and detect the stage of cancer. However, this combination leads to several errors, such as:
  – Misdiagnosis of cancer or even its stage results from an unknown spatial shift between the PET and CT images. For example, it is difficult to identify whether a tumor in the lung is pleural, close to the chest wall, or invasive.
  – If any tissues absorb the radiation out of the PET, a second error occurs. This error is due to the large difference in attenuation, which is diverted to correct the attenuation, such as the skin’s surface or the upper part of the liver.
- **HIs Images:**
  Handling gray-scale images is considered a challenging issue of HIs. This can be handled with certain processes, such as the transformation between the color spaces, the change in the image sizes to adapt to the GPU, and the change in the resolution of the images [16]. There are other challenges in the analysis process of HIs, such as a few images to train, the representation of feature complexity, and the size of the HI being extremely large [125,126]. These challenges are briefly discussed in the following points:
  – The variance in histopathology from one cancer case to another points to a tangible variance in distributions of color, texture, scale, and morphology [127]. This makes it challenging to find a clear and consistent structure for the diagnosis of cancer and for all cases of cancer. Hence, one of the primary functions in high-level MIA is the feature representation as well as classification and segmentation.
  – The size of HIs is huge. This grows the database of this modality of image and, therefore, the computation complexity, making it a challenge to analyze the images. For a 100,000 × 100,000 pixel image, a full scan of the histopathology part of the model can be performed. This modality also includes two million objects. Generally, for every patient, 12 to 20 screen images are performed under the pathological section process. Furthermore, because the database range is large for HIs, a good model with high efficiency both in memory and time is required, and the algorithm for learning should be able to obtain a large amount of data from it [128,129].

### 5.2. Challenges Related to Datasets

Many publicly available datasets used to diagnose many diseases, particularly colon cancer, are briefly discussed here. Finally, DL and ML techniques help identify cancer in nearly stage, leading to early treatment and a lower mortality rate than after symptom development. Additionally, you can prevent colorectal cancer before it progresses by removing nonmalignant tissues, which can be achieved through screening tests to make it easier to diagnose the disease. Many challenges have arisen and are summarized in the following points:

- The image size is the first obstacle if it is little or not enough to train, specifically in the medical field.
- The medical image database is often small, making the available applications that use ML algorithms inefficient for handling new medical images.
- The process of collecting medical images is expensive due to the two following factors: [130]:
  1. The first is that the injury rate has decreased recently, and the size of MIs is associated with the number of injury cases directly; thus, it is also difficult to obtain the images.
  2. The other purpose is that MIs require manual feedback for clarity, which requires a great deal of effort. Furthermore, manual comments can also be vague, even if experts write them.

## 6. Future Research Directions

To detect and prevent colon cancer, the gold standard is colonoscopy. According to the reviewed research, often half of the studies contained reviews of colonoscopy, including polyp classification, detection, and segmentation. There are few studies on WCE that are based on endoscopy while there are many based on colonoscopy.

In the diagnosis process of colon cancer, colonoscopy is the main and common standard method used to confirm these data. Recently, studies are moving towards technology, such as DL, and studying or relying on the field of ML. DL can be utilized as a secondary tool for screening but not as an alternative for a specialized endoscopist. DL can also be used in the detection process of missed cells by maximizing the quality of endoscopists.In future work, researchers should consider the following research directions:

- MIA is an active topic to research in the ML area. For colorectal cancer screening using the common HI modality, it can be handled with certain processes, such as:
  – The transformation between the color spaces and the change resolution of the images to adapt them to the GPU.
  – The change in the resolution of the images to decrease the computation complexity, making it less complicated to analyze.
  – We need a consistent and clear of features definition due to the histopathology difference from one cancer stage to another. This results in a significant distribution difference of the color texture, morphology, and scale as well as segmentation and classification.
- We need available applications and platforms that use ML and DL algorithms efficiently for handling new medical images.
- A retroactive study in more than one institute.
- Applying the emerging DL techniques, such as transfer learning, autoencoders, and generative adversarial networks.

## 7. Conclusions

Generally, after diagnosing the presence of cancer, the next most important step is diagnosing the cancer stage, since an appropriate choice of treatment method and duration depends on this information. Identifying the cancer stage or grade mainly depends on analyzing the structure of the tissue region, which is performed using various tests for screening that can be utilized to explore polyps or colorectal cancer in images. This paper presented a comprehensive review regarding the diagnosis process for colon cancer. We presented different image modalities used for the analysis process.

The most common method used for colon cancer is HIs, which are captured by a microscope. The current state-of-the-art techniques (classified into ML and DL techniques) were reviewed, as these help to determine early-stage cancer, thus, leading to early treatment and lowering the mortality rate. These techniques can also aid in inhibiting the progress of colorectal cancer through the removal of nonmalignant cells after using screening tests to diagnosis the disease early.

Regarding the scientific efforts on this research topic, we also introduced many future research techniques to be investigated. In the future, we intend to implement a comparative study of the most prominent ML and DL techniques in a unified environment using a set of benchmark datasets and predefined evaluation metrics.

## Figures and Tables

**Table 1:** Comparison between symptoms of tumor spread across various organs at the fourth stage.

No.	Spread to Various Organs	Symptoms
1	Liver	Pain on the right side of the abdomen. Constant feeling of illness and fatigue. Loss of weight and appetite. Abdominal bulge due to fluid assembly. Itching disorders of the skin.
2	Lung	Constant cough. Shortness of breath. Duplicate infections in the lungs. Bloody cough. Fluid assembly around the lung.
3	Bone	Pain in injured bones. Bone weakness and increased risk of fracture.
4	Lymph nodes	Swollen lymph node.

**Table 2:** A list of abbreviations with the corresponding definitions used in this survey.

Abbreviation	Definition	Abbreviation	Definition
ML	Machine Learning	DL	Deep Learning
ACS	American Cancer Society	MIA	Medical Image Analysis
AI	Artificial Intelligence	DIP	Digital Image Processing
HC	Hand Crafted	CT	Computed Tomography
ERUS	Endorectal Ultrasound	CTC	Computed Tomography Colonoscopy
PET	Positron Emission Tomography	CRC	Colorectal Cancer
CAD	Computer-Aided Diagnosis	CBIR	Content Based Image Retrieval
PACs	Polycyclic Aromatic Compounds	WSIs	Whole-Slide Images
CNN	Convolutional Neural Network	MMR	Mismatch Repair
PCNSL	Primary Central Nervous System Lymphomas	FDG	Fluoro-2-Deoxy-d-Glucose
GlaS	Gland Segmentation	KID	Kent Integrated Dataset
NBI	Narrow-Band Imaging	TP	True Positive
FP	False Positive	TN	True Negative
FN	False Negative	FPR	False positive rate
PPV	Positive Predictive Value	ROC	Receiver Operating Characteristic
H&E	Hematoxylin Eosin	LBP	Local Binary Patterns
SIFT	Scale Invariant Feature Transform	HOG	Histogram Of Gradient
GBM	Glioblastoma	SVM	Support Vector Machine
OMIS	Opto-magnetic Imaging Spectro-scop	WCE	Wireless Capsule Endo-scop
ROI	Region of Interest	SGLDM	Spatial Gray Level Dependence Matrices
2DReCA	Two-dimensional entropy with a Cultural Algorithm	KNN	K-Nearest Neighbor
HSI	Hyper-Spectral Imaging	LDA	Linear Discriminant Analysis
MLP	Multi-Layer Perceptron	RF	Random Forest
GLCM	Gray Level Co-occurrence Matrix	ANN	Artificial Neural Network
QDA	Quadratic Discriminant Analysis	CDT	Decision Tree
NLP	Natural Language Processing	TCGA	The Cancer Genome Atlas
R-CNN	Region-based Convolutional Neural Network	SSD	Single Shot Multi-Box Detector
RNN	Recurrent Neural Network	PCA	Principal Component Analysis

**Table 3:** Academic databases.

No.	Academic Journals	Link (Accessed on 1 November 2022)
1	MDPI	https://www.mdpi.com/
2	IEEE Explore	https://ieeexplore.ieee.org/Xplore/home.jsp
3	HINDAWI	https://www.hindawi.com/
4	ELSEVIER	https://www.elsevier.com/en-xm
5	SPRINGER	https://jast-journal.springeropen.com/
6	NATURE	https://www.nature.com/
7	THE SCIENCE AND INFORMATION	https://thesai.org/
8	FRONTIERS	https://www.frontiersin.org/

**Table 6:** Summary of ML-based studies for colorectal cancer diagnosis.

Author (Year)	Imaging Modality	Pre-Processing	Feature Extraction	Classification	Dataset	Results
**Niazi et al. [81] (2019)**	Microscopy Images	Stain Normalization, Contrast enhancement using Limited Adaptive Histogram Equalization	K-means, clustering	MLP, SVM, KNNs, Kernel Discriminant model	CRCHisto-Phenotypes	Accuracy: 99.8%
**Rasti et al. [82] (2019)**	Endomicroscopy Images	mean, standard deviation, variance, and the skewness of the raw pixel values	clustering	SVM	ImageNet/ILSVRC	Accuracy: 99.93%
**Na et al. [83,84,85] (2019)**	Neuro-imaging	SMOTE technique	GBM	Decision tree classifier	KLoSA	Sensitivity: 96.7% Specificity: 0.825 AUC: 92.1%
**Rathore et al. [86] (2019)**	Colon tissue histology images	Segmentation using	Qualification of tissue morphology on the basis of image, local, and gland features	Detection: RBF kernel of SVM Classification: using majority voting based on the predictions of linear, RBF, and sigmoid kernel of SVM.	-GlaS, -RMC	For Detection (Train GlaS − Test RMC = 93.7%, Train RMC − Test GlaS = 94.5%), For Classification: (Train GlaS −Test RMC = 95%, Train RMC − Test GlaS = 95%)
**Dragicevic et al. [87] (2019)**	Opto-magnetic Imaging Spectroscopy (OMIS)	Spectral image processing	N/A	Detection: Multilayer Perceptron Neural Network and Naïve Bayes	The First Surgical Clinic, Clinical Centre of Belgrade, Serbia.	Accuracy of 92.59% using Multilayer Perceptron Neural Network Accuracy of 89.87% using Naïve Bayes
**Sundaram et al. [88] (2019)**	Wireless Capsule Endoscopy (WCE)	Weiner filtering, ROI-based color histogram	Segmentation: K-means clustering Feature Extraction: Spatial gray level dependence matrices (SGLDM)	Detection: SVM Classification: SVM	N/A	Sensitivity 96% Specificity 95.4% Accuracy 95.7%
**Babu et al. [89] (2020)**	Colon biopsy images	Stain Normalization. Contrast enhancement using Limited Adaptive Histogram Equalization.	Segmentation: 2DReCA entropy-based thresholding Feature Extraction: Shape and texture descriptors.	A random forest classifier	GlaS.	Accuracy of: dataset A 98.50% dataset B 96.48% dataset C 95% dataset D 96%
**Fahami et al. [90] (2021)**	HIs	Normalization, Dimension reduction	Clustering	KNNs, Decision Tree	HTSeq-FPKM-UQ	Accuracy: 97.49 ± 2.92% Percision: 100.0 ± 90.00% Recall: 95.00 ± 5.83%
**Jansen-Winkeln et al. [91] (2021)**	Hyper-spectral imaging (HSI)	Image smoothing using Savitzky–Golay filter normalization, balancing using down-sampling	N/A	Detection: Multilayer Perceptron Neural Network, SVM, and RF.	Paper-specific dataset	Sensitivity of 86% Specificity of 95% With Multilayer Perceptron Neural Network
**Talukder et al. [92] (2022)**	HIs.	Image Resizing Feature scaling	Feature Extraction: Transfer learning, such as VGG16, VGG19, DenseNet169, DenseNet201	RF, SVM, LR, MLP, XGB, and LGB	LC25000	Accuracy of colon: 99.05%, lung: 100%, colon and lung: 99.30%
**Chehade et al. [93] (2022)**	HIs.	Unsharp masking, Stain normalization	Feature Extraction: First order statistics GLCM, Hu invariant moments Feature Selection: Recursive feature elimination (RFE)	SVM RF, XGBoost LightGBM, LDA, MLP	LC25000	F1-score of 98.8%, Accuracy of 99%
**Alqudah et al. [94] (2022)**	HIs.	Represent input images in three different color spaces: RGB, HSV, and L*A*B* color spaces	Feature Extraction: 3D GLCM of RGB, HSV, and L*A*B	SVM, ANN, KNN, QDA, and CDT.	Multi-class colorectal histology images	Accuracy and sensitivity using RGB color space:QDA: 97.32, 97.30KNN: 92.10, 91.71SVM: 94.68, 94.62ANN: 92.58, 92.40CDT: 91.92, 91.75

**Table 7:** General strengths and limitations of ML studies.

Author (Year)	Strengths	Limitations
**Rasti et al. [82] (2019)**	The proposed approach produced results of 98.49%, which were applied for the more difficult first case, in addition to the results of a 99.93% positive rate acquired for the second scheme.	The database size is not compatible with other domains in machine learning. They should address the computational complexity required for different stages of segmentation, detection, and classification.
**Rathore et al. [86] (2019)**	They evaluated their model with two datasets for colon cancer by achieving the performance of each one in addition to ensemble classifiers. This methodology is reliable for broader applicability across diverse clinical settings.	A suitable feature-selection technique should be employed for reducing redundant features. They should address the computational complexity required for different stages of segmentation, detection, and classification.
**Sundaram et al. [88] (2019)**	This model for enhancing the approach OF CAD with low complexity time. Can be used to detect early stages of colon cancer for patients.	This approach needs to be validated on different benchmark datasets. The performance of the WCE screening method should be compared with different screening methods.
**Babu et al. [89] (2020)**	Their model supplies superior results for the segmentation for different image modalities, irrespective of different magnification levels. The showed approach with 2DReCA segmentation and a hybrid features set proved to have acceptable accuracy for classification.	Does not address overlapping cell segmentation and the region of colon gland segmentation for improving precision.
**Fahami et al. [90] (2021)**	Utilized a method for normalization that was efficient more than the others, which enhanced the overall quality of the research.	A few approaches of DL were utilized on data of colon cancer. A few methods of normalization and method of fuzzy clustering.
**Jansen-Winkeln et al. [91] (2021)**	Hyperspectral imaging combined with automatic classification can be used to distinguish between healthy mucosa and CRC. Too many false positive areas were detected. The changes of biological support due to chemotherapy to the cell that can be detected with HSI.	Developing HSI-based systems to perform non-invasive and contactless optical biopsies of in vivo tissue.
**Talukder et al. [92] (2022)**	This model outperformed other techniques and effectively identified different classes of colon and lung cancers.	Further work on image pre-processing is required as noise-free and sharp images will yield discriminative features, which will, in turn, enhance the performance.
**Chehade et al. [93] (2022)**	The authors achieved acceptable performance using machine-learning techniques, from the application of deep-learning methods.	The model performance should be improved by applying different feature extraction techniques
**Alqudah et al. [94] (2022)**	An acceptable performance for classifying colorectal cancer utilized HIs not with gray level texture features but with colored features.	Image preprocessing and segmentation should be addressed before further processing in order to increase the performance.

## Data Availability

Not applicable.

## References

[1] Allison J.E. (2010). Colorectal cancer screening guidelines: The importance of evidence and transparency. Gastroenterology.

[2] An F.P., Liu J.E. (2020). Medical Image Segmentation Algorithm Based on Optimized Convolutional Neural Network-Adaptive Dropout Depth Calculation. Complexity.

[3] Araghi M., Soerjomataram I., Jenkins M., Brierley J., Morris E., Bray F., Arnold M. (2019). Global trends in colorectal cancer mortality: Projections to the year 2035. Int. J. Cancer.

[4] Barish M.A., Soto J.A., Ferrucci J.T. (2005). Consensus on current clinical practice of virtual colonoscopy. Am. J. Roentgenol..

[5] Thun M.J., Calle E.E., Namboodiri M.M., Flanders W.D., Coates R.J., Byers T., Boffetta P., Garfinkel L., Heath C.W. (1992). Risk factors for fatal colon cancer in a large prospective study. JNCI J. Natl. Cancer Inst..

[6] Rathore S., Hussain M., Ali A., Khan A. (2013). A recent survey on colon cancer detection techniques. IEEE/ACM Trans. Comput. Biol. Bioinform..

[7] Baxter N.N., Goldwasser M.A., Paszat L.F., Saskin R., Urbach D.R., Rabeneck L. (2009). Association of colonoscopy and death from colorectal cancer. Ann. Intern. Med..

[8] Bera K., Schalper K.A., Rimm D.L., Velcheti V., Madabhushi A. (2019). Artificial intelligence in digital pathology—New tools for diagnosis and precision oncology. Nat. Rev. Clin. Oncol..

[9] Kitayama J., Nagawa H., Tsuno N., Osada T., Hatano K., Sunami E., Saito H., Muto T. (1999). Laminin mediates tethering and spreading of colon cancer cells in physiological shear flow. Br. J. Cancer.

[10] Burdan F., Sudol-Szopinska I., Staroslawska E., Kolodziejczak M., Klepacz R., Mocarska A., Caban M., Zelazowska-Cieslinska I., Szumilo J. (2015). Magnetic resonance imaging and endorectal ultrasound for diagnosis of rectal lesions. Eur. J. Med. Res..

[11] Bychkov D., Linder N., Turkki R., Nordling S., Kovanen P.E., Verrill C., Walliander M., Lundin M., Haglund C., Lundin J. (2018). Deep learning based tissue analysis predicts outcome in colorectal cancer. Sci. Rep..

[12] Levin B., Lieberman D.A., McFarland B., Andrews K.S., Brooks D., Bond J., Dash C., Giardiello F.M., Glick S., Johnson D. (2008). Screening and surveillance for the early detection of colorectal cancer and adenomatous polyps, 2008: A joint guideline from the American Cancer Society, the US Multi-Society Task Force on Colorectal Cancer, and the American College of Radiology. Gastroenterology.

[13] Chaddad A., Tanougast C., Dandache A., Al Houseini A., Bouridane A. Improving of colon cancer cells detection based on Haralick’s features on segmented histopathological images. Proceedings of the 2011 IEEE International Conference on Computer Applications and Industrial Electronics (ICCAIE).

[14] Hur C., Chung D.C., Schoen R.E., Gazelle G.S. (2007). The management of small polyps found by virtual colonoscopy: Results of a decision analysis. Clin. Gastroenterol. Hepatol..

[15] Gunduz-Demir C., Kandemir M., Tosun A.B., Sokmensuer C. (2010). Automatic segmentation of colon glands using object-graphs. Med. Image Anal..

[16] Wang D., Foran D.J., Ren J., Zhong H., Kim I.Y., Qi X. Exploring automatic prostate histopathology image gleason grading via local structure modeling. Proceedings of the 2015 37th Annual International Conference of the IEEE Engineering in Medicine and Biology Society (EMBC).

[17] Dal Molin M., Matthaei H., Wu J., Blackford A., Debeljak M., Rezaee N., Wolfgang C.L., Butturini G., Salvia R., Bassi C. (2013). Clinicopathological correlates of activating GNAS mutations in intraductal papillary mucinous neoplasm (IPMN) of the pancreas. Ann. Surg. Oncol..

[18] Masud M., Sikder N., Nahid A.A., Bairagi A.K., AlZain M.A. (2021). A machine learning approach to diagnosing lung and colon cancer using a deep learning-based classification framework. Sensors.

[19] Demir C., Yener B. (2005). Automated Cancer Diagnosis Based on Histopathological Images: A Systematic Survey.

[20] Pacal I., Karaboga D., Basturk A., Akay B., Nalbantoglu U. (2020). A comprehensive review of deep learning in colon cancer. Comput. Biol. Med..

[21] Davri A., Birbas E., Kanavos T., Ntritsos G., Giannakeas N., Tzallas A.T., Batistatou A. (2022). Deep Learning on Histopathological Images for Colorectal Cancer Diagnosis: A Systematic Review. Diagnostics.

[22] Elazab N., Soliman H., El-Sappagh S., Islam S., Elmogy M. (2020). Objective Diagnosis for Histopathological Images Based on Machine Learning Techniques: Classical Approaches and New Trends. Mathematics.

[23] Bar-Shalom R., Valdivia A.Y., Blaufox M.D. (2000). PET imaging in oncology. Semin. Nucl. Med..

[24] DeBarros M., Steele S.R. (2013). Colorectal cancer screening in an equal access healthcare system. J. Cancer.

[25] Horton K.M., Abrams R.A., Fishman E.K. (2000). Spiral CT of colon cancer: Imaging features and role in management. Radiographics.

[26] Ding H., Pan Z., Cen Q., Li Y., Chen S. (2020). Multi-scale fully convolutional network for gland segmentation using three-class classification. Neurocomputing.

[27] Hartmann D., Bassler B., Schilling D., Adamek H.E., Jakobs R., Pfeifer B., Eickhoff A., Zindel C., Riemann J.F., Layer G. (2006). Colorectal polyps: Detection with dark-lumen MR colonography versus conventional colonoscopy. Radiology.

[28] Kekelidze M., D’Errico L., Pansini M., Tyndall A., Hohmann J. (2013). Colorectal cancer: Current imaging methods and future perspectives for the diagnosis, staging and therapeutic response evaluation. World J. Gastroenterol..

[29] Esgiar A.N., Naguib R.N., Sharif B.S., Bennett M.K., Murray A. (2002). Fractal analysis in the detection of colonic cancer images. IEEE Trans. Inf. Technol. Biomed..

[30] Geiger T.M., Ricciardi R. (2009). Screening options and recommendations for colorectal cancer. Clin. Colon Rectal Surg..

[31] Rathore S., Iftikhar M.A., Hussain M., Jalil A. Classification of colon biopsy images based on novel structural features. Proceedings of the 2013 IEEE ninth International Conference on Emerging Technologies (ICET).

[32] Li J., Ye G., Das A., Zhao R., Gong Y. Advancing acoustic-to-word CTC model. Proceedings of the 2018 IEEE International Conference on Acoustics, Speech and Signal Processing (ICASSP).

[33] Lustig M., Donoho D.L., Santos J.M., Pauly J.M. (2008). Compressed sensing MRI. IEEE Signal Process. Mag..

[34] Nerad E., Lambregts D.M., Kersten E.L., Maas M., Bakers F.C., van den Bosch H.C., Grabsch H.I., Beets-Tan R.G., Lahaye M.J. (2017). MRI for local staging of colon cancer: Can MRI become the optimal staging modality for patients with colon cancer?. Dis. Colon Rectum.

[35] Hanasono M.M., Kunda L.D., Segall G.M., Ku G.H., Terris D.J. (1999). Uses and limitations of FDG positron emission tomography in patients with head and neck cancer. Laryngoscope.

[36] Sena P., Fioresi R., Faglioni F., Losi L., Faglioni G., Roncucci L. (2019). Deep learning techniques for detecting preneoplastic and neoplastic lesions in human colorectal histological images. Oncol. Lett..

[37] Noorbakhsh J., Farahmand S., Namburi S., Caruana D., Rimm D., Soltanieh-ha M., Zarringhalam K., Chuang J.H. (2020). Deep learning-based cross-classifications reveal conserved spatial behaviors within tumor histological images. Nat. Commun..

[38] Babu T., Singh T., Gupta D., Hameed S. (2021). Colon cancer prediction on histological images using deep learning features and Bayesian optimized SVM. J. Intell. Fuzzy Syst..

[39] Goggi J.L., Hartimath S.V., Xuan T.Y., Khanapur S., Jieu B., Chin H.X., Ramasamy B., Cheng P., Rong T.J., Fong Y.F. (2021). Granzyme B PET Imaging of Combined Chemotherapy and Immune Checkpoint Inhibitor Therapy in Colon Cancer. Mol. Imaging Biol..

[40] Salvatore L., Imperatori M., Arnoldi E., Carnaghi C., Cordio S., Cosimelli M., Cremolini C., Maiello E., Martinelli E., Normanno N. (2020). Management of patients with early-stage colon cancer: Guidelines of the Italian Medical Oncology Association. ESMO Open.

[41] Hodolic M., Ambrosini V., Fanti S. (2020). Potential use of radiolabelled neurotensin in PET imaging and therapy of patients with pancreatic cancer. Nucl. Med. Commun..

[42] Godkhindi A.M., Gowda R.M. Automated detection of polyps in CT colonography images using deep learning algorithms in colon cancer diagnosis. Proceedings of the 2017 International Conference on Energy, Communication, Data Analytics and Soft Computing (ICECDS).

[43] Mukai K., Ishida Y., Okajima K., Isozaki H., Morimoto T., Nishiyama S. (2006). Usefulness of preoperative FDG-PET for detection of gastric cancer. Gastric Cancer.

[44] Moroz M.A., Kochetkov T., Cai S., Wu J., Shamis M., Nair J., De Stanchina E., Serganova I., Schwartz G.K., Banerjee D. (2011). Imaging colon cancer response following treatment with AZD1152: A preclinical analysis of [18F] fluoro-2-deoxyglucose and fluorothymidine imaging. Clin. Cancer Res..

[45] Kalkan H., Nap M., Duin R.P., Loog M. Automated classification of local patches in colon histopathology. Proceedings of the Proceedings of the 21st International Conference on Pattern Recognition (ICPR2012).

[46] Greenspan H., Van Ginneken B., Summers R.M. (2016). Guest editorial deep learning in medical imaging: Overview and future promise of an exciting new technique. IEEE Trans. Med. Imaging.

[47] Doi K. (2007). Computer-aided diagnosis in medical imaging: Historical review, current status and future potential. Comput. Med. Imaging Graph..

[48] Hamilton P.W., Bartels P.H., Thompson D., Anderson N.H., Montironi R., Sloan J.M. (1997). Automated location of dysplastic fields in colorectal histology using image texture analysis. J. Pathol. J. Pathol. Soc. Great Br. Irel..

[49] Jia X., Xing X., Yuan Y., Xing L., Meng M.Q.H. (2019). Wireless capsule endoscopy: A new tool for cancer screening in the colon with deep-learning-based polyp recognition. Proc. IEEE.

[50] Hou L., Agarwal A., Samaras D., Kurc T.M., Gupta R.R., Saltz J.H. Robust histopathology image analysis: To label or to synthesize?. Proceedings of the IEEE/CVF Conference on Computer Vision and Pattern Recognition.

[51] Cheng A.S., Leung S.C., Gao D., Burugu S., Anurag M., Ellis M.J., Nielsen T.O. (2020). Mismatch repair protein loss in breast cancer: Clinicopathological associations in a large British Columbia cohort. Breast Cancer Res. Treat..

[52] Kim H.O., Kim J.S., Kim S.O., Chae S.Y., Oh S.J., Seo M., Lee S.H., Oh J.S., Ryu J.S., Huh J.r. (2020). Clinicopathological characteristics of primary central nervous system lymphoma with low 18F-fludeoxyglucose uptake on brain positron emission tomography. Medicine.

[53] Janowczyk A., Madabhushi A. (2016). Deep learning for digital pathology image analysis: A comprehensive tutorial with selected use cases. J. Pathol. Inform..

[54] Burt R.W. (2010). Strategies for colon cancer screening with considerations of cost and access to care. J. Natl. Compr. Cancer Netw..

[55] Fakoor R., Ladhak F., Nazi A., Huber M. Using deep learning to enhance cancer diagnosis and classification. Proceedings of the International Conference on Machine Learning.

[56] Awan R., Sirinukunwattana K., Epstein D., Jefferyes S., Qidwai U., Aftab Z., Mujeeb I., Snead D., Rajpoot N. (2017). Glandular morphometrics for objective grading of colorectal adenocarcinoma histology images. Sci. Rep..

[57] Gamper J., Koohbanani N.A., Benes K., Graham S., Jahanifar M., Khurram S.A., Azam A., Hewitt K., Rajpoot N. (2020). Pannuke dataset extension, insights and baselines. arXiv.

[58] Sirinukunwattana K., Snead D.R., Rajpoot N.M. (2015). A stochastic polygons model for glandular structures in colon histology images. IEEE Trans. Med. Imaging.

[59] Graham S., Vu Q.D., Raza S.E.A., Azam A., Tsang Y.W., Kwak J.T., Rajpoot N. (2019). Hover-net: Simultaneous segmentation and classification of nuclei in multi-tissue histology images. Med. Image Anal..

[60] Jha D., Smedsrud P.H., Riegler M.A., Halvorsen P., de Lange T., Johansen D., Johansen H.D. Kvasir-seg: A segmented polyp dataset. Proceedings of the International Conference on Multimedia Modeling.

[61] Sirinukunwattana K., Raza S.E.A., Tsang Y.W., Snead D.R., Cree I.A., Rajpoot N.M. (2016). Locality sensitive deep learning for detection and classification of nuclei in routine colon cancer histology images. IEEE Trans. Med. Imaging.

[62] Lewer D., Bourne T., George A., Abi-Aad G., Taylor C., George J. (2018). Data Resource: The Kent Integrated Dataset (KID). Int. J. Popul. Data Sci..

[63] Mesejo P., Pizarro D., Abergel A., Rouquette O., Beorchia S., Poincloux L., Bartoli A. (2016). Computer-aided classification of gastrointestinal lesions in regular colonoscopy. IEEE Trans. Med. Imaging.

[64] Shaban M., Awan R., Fraz M.M., Azam A., Tsang Y.W., Snead D., Rajpoot N.M. (2020). Context-aware convolutional neural network for grading of colorectal cancer histology images. IEEE Trans. Med. Imaging.

[65] Pogorelov K., Randel K.R., Griwodz C., Eskeland S.L., de Lange T., Johansen D., Spampinato C., Dang-Nguyen D.T., Lux M., Schmidt P.T. Kvasir: A multi-class image dataset for computer aided gastrointestinal disease detection. Proceedings of the eighth ACM on Multimedia Systems Conference.

[66] Leenhardt R., Li C., Le Mouel J.P., Rahmi G., Saurin J.C., Cholet F., Boureille A., Amiot X., Delvaux M., Duburque C. (2020). CAD-CAP: A 25,000-image database serving the development of artificial intelligence for capsule endoscopy. Endosc. Int. Open.

[67] Bernal J., Tajkbaksh N., Sanchez F.J., Matuszewski B.J., Chen H., Yu L., Angermann Q., Romain O., Rustad B., Balasingham I. (2017). Comparative validation of polyp detection methods in video colonoscopy: Results from the MICCAI 2015 endoscopic vision challenge. IEEE Trans. Med. Imaging.

[68] Vázquez D., Bernal J., Sánchez F.J., Fernández-Esparrach G., López A.M., Romero A., Drozdzal M., Courville A. (2017). A benchmark for endoluminal scene segmentation of colonoscopy images. J. Healthc. Eng..

[69] Bernal J., Sánchez F.J., Fernández-Esparrach G., Gil D., Rodríguez C., Vilariño F. (2015). WM-DOVA maps for accurate polyp highlighting in colonoscopy: Validation vs. saliency maps from physicians. Comput. Med. Imaging Graph..

[70] Javed S., Mahmood A., Fraz M.M., Koohbanani N.A., Benes K., Tsang Y.W., Hewitt K., Epstein D., Snead D., Rajpoot N. (2020). Cellular community detection for tissue phenotyping in colorectal cancer histology images. Med. Image Anal..

[71] Kather J.N., Krisam J., Charoentong P., Luedde T., Herpel E., Weis C.A., Gaiser T., Marx A., Valous N.A., Ferber D. (2019). Predicting survival from colorectal cancer histology slides using deep learning: A retrospective multicenter study. PLoS Med..

[72] Kainz P., Pfeiffer M., Urschler M. (2017). Segmentation and classification of colon glands with deep convolutional neural networks and total variation regularization. PeerJ.

[73] Puig D. (2020). Assessing the Impact of a Preprocessing Stage on Deep Learning Architectures for Breast Tumor Multi-class Classification with Histopathological Images. Proceedings of the High Performance Computing: Sixth Latin American Conference, CARLA 2019.

[74] Jang H.J., Lee A., Kang J., Song I.H., Lee S.H. (2020). Prediction of clinically actionable genetic alterations from colorectal cancer histopathology images using deep learning. World J. Gastroenterol..

[75] Kaminski M.F., Regula J., Kraszewska E., Polkowski M., Wojciechowska U., Didkowska J., Zwierko M., Rupinski M., Nowacki M.P., Butruk E. (2010). Quality indicators for colonoscopy and the risk of interval cancer. N. Engl. J. Med..

[76] Khatun R., Chatterjee S. Machine learning approach for segmenting glands in colon histology images using local intensity and texture features. Proceedings of the 2018 IEEE eighth International Advance Computing Conference (IACC).

[77] Constantinescu A.F., Ionescu M., Rogoveanu I., Ciurea M.E., Streba C.T., Iovanescu V.F., Artene S.A., Vere C.C. (2015). Analysis of wireless capsule endoscopy images using local binary patterns. Appl. Med. Inform..

[78] Alwan M.G., AL-Brazinji S.M., Mosslah A.A. (2022). Automatic panoramic medical image stitching improvement based on feature-based approach. Period. Eng. Nat. Sci..

[79] Wargnier-Dauchelle V., Chane C.S., Histace A. Saliency maps of video-colonoscopy images for the analysis of their content and the prevention of colorectal cancer risks. Proceedings of the Biosignals 2020.

[80] Kieffer B., Babaie M., Kalra S., Tizhoosh H.R. Convolutional neural networks for histopathology image classification: Training vs. using pre-trained networks. Proceedings of the 2017 Seventh International Conference on Image Processing Theory, Tools and Applications (IPTA).

[81] Niazi M.K.K., Parwani A.V., Gurcan M.N. (2019). Digital pathology and artificial intelligence. Lancet Oncol..

[82] Rasti P., Wolf C., Dorez H., Sablong R., Moussata D., Samiei S., Rousseau D. (2019). Machine Learning-Based Classification of the Health State of Mice Colon in Cancer Study from Confocal Laser Endomicroscopy. Sci. Rep..

[83] Na K.S. (2019). Prediction of future cognitive impairment among the community elderly: A machine-learning based approach. Sci. Rep..

[84] Singh S.P., Janjuha S., Chaudhuri S., Reinhardt S., Kränkel A., Dietz S., Eugster A., Bilgin H., Korkmaz S., Zararsız G. (2018). Machine learning based classification of cells into chronological stages using single-cell transcriptomics. Sci. Rep..

[85] Min X., Yu B., Wang F. (2019). Predictive modeling of the hospital readmission risk from patients’ claims data using machine learning: A case study on COPD. Sci. Rep..

[86] Rathore S., Iftikhar M.A., Chaddad A., Niazi T., Karasic T., Bilello M. (2019). Segmentation and grade prediction of colon cancer digital pathology images across multiple institutions. Cancers.

[87] Dragicevic A., Matija L., Krivokapic Z., Dimitrijevic I., Baros M., Koruga D. (2019). Classification of healthy and cancer states of colon epithelial tissues using opto-magnetic imaging spectroscopy. J. Med. Biol. Eng..

[88] Shanmuga Sundaram P., Santhiyakumari N. (2019). An enhancement of computer aided approach for colon cancer detection in WCE images using ROI based color histogram and SVM2. J. Med. Syst..

[89] Babu T., Singh T., Gupta D. (2020). Colon cancer prediction using 2DR e CA segmentation and hybrid features on histopathology images. IET Image Process..

[90] Fahami M.A., Roshanzamir M., Izadi N.H., Keyvani V., Alizadehsani R. (2021). Detection of effective genes in colon cancer: A machine learning approach. Inform. Med. Unlocked.

[91] Jansen-Winkeln B., Barberio M., Chalopin C., Schierle K., Diana M., Köhler H., Gockel I., Maktabi M. (2021). Feedforward artificial neural network-based colorectal cancer detection using hyperspectral imaging: A step towards automatic optical biopsy. Cancers.

[92] Talukder M.A., Islam M.M., Uddin M.A., Akhter A., Hasan K.F., Moni M.A. (2022). Machine learning-based lung and colon cancer detection using deep feature extraction and ensemble learning. Expert Syst. Appl..

[93] Hage Chehade A., Abdallah N., Marion J.M., Oueidat M., Chauvet P. (2022). Lung and colon cancer classification using medical imaging: A feature engineering approach. Phys. Eng. Sci. Med..

[94] Alqudah A.M., Alqudah A. (2022). Improving machine learning recognition of colorectal cancer using 3D GLCM applied to different color spaces. Multimed. Tools Appl..

[95] Deng J., Dong W., Socher R., Li L.J., Li K., Fei-Fei L. Imagenet: A large-scale hierarchical image database. Proceedings of the 2009 IEEE Conference on Computer Vision and Pattern Recognition.

[96] Sutskever I., Vinyals O., Le Q.V. (2014). Sequence to sequence learning with neural networks. Adv. Neural Inf. Process. Syst..

[97] Sarraf S., Tofighi G. Deep learning-based pipeline to recognize Alzheimer’s disease using fMRI data. Proceedings of the 2016 Future Technologies Conference (FTC).

[98] Shan H., Padole A., Homayounieh F., Kruger U., Khera R.D., Nitiwarangkul C., Kalra M.K., Wang G. (2019). Competitive performance of a modularized deep neural network compared to commercial algorithms for low-dose CT image reconstruction. Nat. Mach. Intell..

[99] Liu Y., Gadepalli K., Norouzi M., Dahl G.E., Kohlberger T., Boyko A., Venugopalan S., Timofeev A., Nelson P.Q., Corrado G.S. (2017). Detecting cancer metastases on gigapixel pathology images. arXiv.

[100] Min J.K., Kwak M.S., Cha J.M. (2019). Overview of deep learning in gastrointestinal endoscopy. Gut Liver.

[101] Shapcott M., Hewitt K.J., Rajpoot N. (2019). Deep learning with sampling in colon cancer histology. Front. Bioeng. Biotechnol..

[102] Esgiar A.N., Naguib R.N., Sharif B.S., Bennett M.K., Murray A. (1998). Microscopic image analysis for quantitative measurement and feature identification of normal and cancerous colonic mucosa. IEEE Trans. Inf. Technol. Biomed..

[103] de Almeida Thomaz V., Sierra-Franco C.A., Raposo A.B. Training data enhancements for robust polyp segmentation in colonoscopy images. Proceedings of the 2019 IEEE 32nd International Symposium on Computer-Based Medical Systems (CBMS).

[104] Kang J., Gwak J. (2019). Ensemble of instance segmentation models for polyp segmentation in colonoscopy images. IEEE Access.

[105] Sornapudi S., Meng F., Yi S. (2019). Region-based automated localization of colonoscopy and wireless capsule endoscopy polyps. Appl. Sci..

[106] Zhang X., Chen F., Yu T., An J., Huang Z., Liu J., Hu W., Wang L., Duan H., Si J. (2019). Real-time gastric polyp detection using convolutional neural networks. PLoS ONE.

[107] Wittenberg T., Zobel P., Rathke M., Mühldorfer S. (2019). Computer aided detection of polyps in whitelight-colonoscopy images using deep neural networks. Curr. Dir. Biomed. Eng..

[108] Ma Y., Li Y., Yao J., Chen B., Deng J., Yang X. Polyp location in colonoscopy based on deep learning. Proceedings of the 2019 eighth international symposium on next generation electronics (ISNE).

[109] Blanes-Vidal V., Baatrup G., Nadimi E.S. (2019). Addressing priority challenges in the detection and assessment of colorectal polyps from capsule endoscopy and colonoscopy in colorectal cancer screening using machine learning. Acta Oncol..

[110] Wang P., Berzin T.M., Brown J.R.G., Bharadwaj S., Becq A., Xiao X., Liu P., Li L., Song Y., Zhang D. (2019). Real-time automatic detection system increases colonoscopic polyp and adenoma detection rates: A prospective randomised controlled study. Gut.

[111] Yuan Y., Qin W., Ibragimov B., Zhang G., Han B., Meng M.Q.H., Xing L. (2019). Densely connected neural network with unbalanced discriminant and category sensitive constraints for polyp recognition. IEEE Trans. Autom. Sci. Eng..

[112] Jia X., Mai X., Cui Y., Yuan Y., Xing X., Seo H., Xing L., Meng M.Q.H. (2020). Automatic polyp recognition in colonoscopy images using deep learning and two-stage pyramidal feature prediction. IEEE Trans. Autom. Sci. Eng..

[113] Tripathi S., Singh S.K. (2020). Ensembling handcrafted features with deep features: An analytical study for classification of routine colon cancer histopathological nuclei images. Multimed. Tools Appl..

[114] Nadimi E.S., Buijs M.M., Herp J., Kroijer R., Kobaek-Larsen M., Nielsen E., Pedersen C.D., Blanes-Vidal V., Baatrup G. (2020). Application of deep learning for autonomous detection and localization of colorectal polyps in wireless colon capsule endoscopy. Comput. Electr. Eng..

[115] Mostafiz R., Hasan M., Hossain I., Rahman M.M. (2020). An intelligent system for gastrointestinal polyp detection in endoscopic video using fusion of bidimensional empirical mode decomposition and convolutional neural network features. Int. J. Imaging Syst. Technol..

[116] Ozawa T., Ishihara S., Fujishiro M., Kumagai Y., Shichijo S., Tada T. (2020). Automated endoscopic detection and classification of colorectal polyps using convolutional neural networks. Ther. Adv. Gastroenterol..

[117] Hamida A.B., Devanne M., Weber J., Truntzer C., Derangère V., Ghiringhelli F., Forestier G., Wemmert C. (2021). Deep learning for colon cancer histopathological images analysis. Comput. Biol. Med..

[118] Liew W.S., Tang T.B., Lin C.H., Lu C.K. (2021). Automatic colonic polyp detection using integration of modified deep residual convolutional neural network and ensemble learning approaches. Comput. Methods Programs Biomed..

[119] Sikder J., Das U.K., Chakma R.J. (2021). Supervised learning-based cancer detection. Int. J. Adv. Comput. Sci. Appl..

[120] Wang W., Tian J., Zhang C., Luo Y., Wang X., Li J. (2020). An improved deep learning approach and its applications on colonic polyp images detection. BMC Med. Imaging.

[121] Tellez D., Litjens G., Bándi P., Bulten W., Bokhorst J.M., Ciompi F., van der Laak J. (2019). Quantifying the effects of data augmentation and stain color normalization in convolutional neural networks for computational pathology. Med. Image Anal..

[122] Tjoa M.P., Krishnan S.M. (2003). Feature extraction for the analysis of colon status from the endoscopic images. Biomed. Eng. Online.

[123] van Roon A.H., van Dam L., Spaander M.C., Lansdorp-Vogelaar I., Dekker E., van Leerdam M.E. (2016). Different modalities for colorectal cancer screening: Experiences in The Netherlands so far. Color. Cancer.

[124] Griffeth L.K. (2005). Use of PET/CT scanning in cancer patients: Technical and practical considerations. Proc. (Bayl. Univ. Med. Cent.).

[125] Winawer S.J., Zauber A.G., Ho M.N., O’brien M.J., Gottlieb L.S., Sternberg S.S., Waye J.D., Schapiro M., Bond J.H., Panish J.F. (1993). Prevention of colorectal cancer by colonoscopic polypectomy. N. Engl. J. Med..

[126] Xu Y., Jia Z., Wang L.B., Ai Y., Zhang F., Lai M., Eric I., Chang C. (2017). Large scale tissue histopathology image classification, segmentation, and visualization via deep convolutional activation features. BMC Bioinform..

[127] Yao Y., Gou S., Tian R., Zhang X., He S. (2021). Automated Classification and Segmentation in Colorectal Images Based on Self-Paced Transfer Network. BioMed Res. Int..

[128] Zauber A.G., van Ballegooijen M., Petitti D., Lansdorp-Vogelaar I. (2010). United States Preventive Services Task Force recommendations: Age to end screening misunderstood. Dis. Colon Rectum.

[129] Zhang X., Liu W., Dundar M., Badve S., Zhang S. (2014). Towards large-scale histopathological image analysis: Hashing-based image retrieval. IEEE Trans. Med. Imaging.

[130] Xu Y., Mo T., Feng Q., Zhong P., Lai M., Eric I., Chang C. Deep learning of feature representation with multiple instance learning for medical image analysis. Proceedings of the 2014 IEEE International Conference on Acoustics, Speech and Signal Processing (ICASSP).

